# Origin Recognition Complex (ORC) Evolution Is Influenced by Global Gene Duplication/Loss Patterns in Eukaryotic Genomes

**DOI:** 10.1093/gbe/evaa011

**Published:** 2020-01-28

**Authors:** Eduard Ocaña-Pallarès, Zaida Vergara, Bénédicte Desvoyes, Manuel Tejada-Jimenez, Ainoa Romero-Jurado, Aurora Galván, Emilio Fernández, Iñaki Ruiz-Trillo, Crisanto Gutierrez

**Affiliations:** e1 Institut de Biologia Evolutiva (CSIC-Universitat Pompeu Fabra), Barcelona, Spain; e2 Centro de Biologia Molecular Severo Ochoa, CSIC-UAM, Cantoblanco, Madrid, Spain; e3 Departamento de Bioquímica y Biología Molecular, Facultad de Ciencias, Universidad de Córdoba, Córdoba, Spain; e4 Departament de Genètica, Microbiologia i Estadística, Universitat de Barcelona, Barcelona, Spain; e5 ICREA, Barcelona, Spain

**Keywords:** origin recognition complex (ORC), DNA replication, eukaryotic evolution, centriole, gene loss, parasitism, whole genome duplication

## Abstract

The conservation of orthologs of most subunits of the origin recognition complex (ORC) has served to propose that the whole complex is common to all eukaryotes. However, various uncertainties have arisen concerning ORC subunit composition in a variety of lineages. Also, it is unclear whether the ancestral diversification of ORC in eukaryotes was accompanied by the neofunctionalization of some subunits, for example, role of ORC1 in centriole homeostasis. We have addressed these questions by reconstructing the distribution and evolutionary history of ORC1-5/CDC6 in a taxon-rich eukaryotic data set. First, we identified ORC subunits previously undetected in divergent lineages, which allowed us to propose a series of parsimonious scenarios for the origin of this multiprotein complex. Contrary to previous expectations, we found a global tendency in eukaryotes to increase or decrease the number of subunits as a consequence of genome duplications or streamlining, respectively. Interestingly, parasites show significantly lower number of subunits than free-living eukaryotes, especially those with the lowest genome size and gene content metrics. We also investigated the evolutionary origin of the ORC1 role in centriole homeostasis mediated by the PACT region in human cells. In particular, we tested the consequences of reducing ORC1 levels in the centriole-containing green alga *Chlamydomonas reinhardtii*. We found that the proportion of centrioles to flagella and nuclei was not dramatically affected. This, together with the PACT region not being significantly more conserved in centriole-bearing eukaryotes, supports the notion that this neofunctionalization of ORC1 would be a recent acquisition rather than an ancestral eukaryotic feature.

## Introduction

DNA replication is essential for the maintenance of the genetic integrity in any cellular lineage. The first event in DNA replication is the specification of potential DNA replication origins (ORIs) by the formation of a stable complex of initiator proteins (Yeeles et al. 2015), a process where the AAA+ ATPases are crucial and a common feature of Bacteria, Archaea, and eukaryotes (Duderstadt and Berger 2008). ORIs in Bacteria and Archaea are marked by the DnaA and the CDC6/ORC1 AAA+ ATPases, respectively (Marques et al. 2016). In eukaryotes, potential ORIs are specified by the formation of prereplication complexes by binding of the origin recognition complex (ORC) and the sequential assembly of Cell Division Cycle 6 (CDC6), CDC10-dependent transcription factor 1 (CDT1), and the minichromosome maintenance (MCM) protein complex (Yeeles et al. 2015). The ORC1, 2, 3, 4, and 5 subunits (ORC1-5) as well as the CDC6 protein possess an AAA+ ATPase domain and presumably evolved from an ancestral CDC6/ORC1 archaeal sequence (Duncker et al. 2009).

Given the presence of orthologs of CDC6 and all ORC1-5 subunits in distantly related lineages such as animals and plants, it was proposed that the whole multiprotein complex was likely to be a conserved feature, common to all eukaryotes (Duncker et al. 2009). However, this scenario was disputed by the finding of a simpler ORC in *Trypanosoma* (Excavata), consisting only of a CDC6/ORC1 protein and hence resembling that of Archaea (Godoy et al. 2009). Indeed, this was proposed as a synapomorphy in favor of an earlier origin of the lineage leading to *Trypanosoma* compared with other eukaryotes (Cavalier-Smith 2010). However, the position of excavates with respect to other eukaryotic groups is still uncertain (Adl et al. 2019). Moreover, other studies revealed the presence of a divergent ORC4 subunit in *Trypanosoma*, and also the absence of ORC subunits in other lineages (Tiengwe et al. 2012; Marques et al. 2016). Thus, it remains unclear whether the whole CDC6+ORC1-5 multiprotein complex was established in a common eukaryotic ancestor, and whether it became highly conserved because its establishment or lost in distinct lineages.

It is also uncertain whether the ancestral diversification of ORC in eukaryotes only involved the subfunctionalization of this molecular complex or whether it was accompanied by the neofunctionalization of some ORC subunits. For example, in humans, the ORC1 subunit is also involved in controlling the centriole and centrosome copy number (Hemerly et al. 2009). ORC1-centrosome interaction is mediated by a pericentrin-AKAP450 centrosomal targeting (PACT) motif located in the C-terminus of the protein and acts independently of the DNA replication function of ORC1 (Hossain and Stillman 2012). Correct targeting of ORC1 to the centrosome is crucial as revealed by deficiencies observed in patients of the Meier-Gorlin syndrome that harbor mutations in the ORC1 gene (Hossain and Stillman 2012). Centrioles, as microtubule organizing complexes (MTOCs), are ancestral in eukaryotes, present in a wide variety of lineages, ranging from animals to green alga (Carvalho-Santos et al. 2011). However, it is unclear whether the role of ORC1 in controlling the integrity of MTOCs is an ancestral eukaryotic feature, or whether it originated in a more recent lineage leading to humans.

We thus addressed all these uncertainties related to ORC evolution by means of a combined bioinformatics and experimental approach. We first reconstructed the distribution and the evolutionary history of CDC6+ORC1-5, the AAA+ ATPase domain-bearing ORC subunits, in a taxon-rich data set (132 eukaryotic proteomes, euk_db). Our orthology detection pipeline allowed us to identify ORC subunits previously thought to be absent in divergent lineages. From this, we reconstructed a series of parsimonious scenarios for the origin of CDC6+ORC1-5 in eukaryotes. Contrary to previous expectations (Duncker et al. 2009), despite the tendency of eukaryotes to preserve the canonical subunit configuration, many lineages increased and decreased the number of subunits as a consequence of whole-genome duplications (WGD) and genome streamlining, respectively. Still, despite the variability observed in the number and losses of subunits, the conservation of either CDC6 or ORC1 appears as an unavoidable constraint in ORC evolution. Finally, we also investigated the potential role of ORC1 in the integrity of MTOC function in *Chlamydomonas reinhardtii*. Algal cells with a reduced expression of CrORC1 did not exhibit unrestricted number of centrioles although they showed differences in the size and the motility of the flagella. Still, these differences are likely to be a consequence of problems in genome replication and cell division, and thus the role of ORC1 in controlling the integrity of the MTOC was probably acquired later on in evolution.

## Materials and Methods

### Orthology Detection Pipeline of ORC Subunits in Eukaryotes

The pipeline used to identify AAA+ ATPase bearing ORC subunit orthologs (CDC6, ORC1, ORC2, ORC3, ORC4, and ORC5; CDC6+ORC1-5) across a data set of 132 eukaryotic species (euk_db, supplementary table 1, Supplementary Material online) consisted in the following steps: 1) Sequence-similarity searches (*BLASTP*, Altschul et al. 1990 and *PfamScan*, Finn et al. 2014) using reference sequences from *Homo sapiens*, *Saccharomyces cerevisiae*, and *Arabidopsis thaliana*. 2) The potential ORC subunits found in the first round were used to find further orthologs that may had remained undetected in euk_db. 3) Phylogenetic reconstruction of CDC6+ORC1-5 using the potential orthologs found in a taxonomically diverse and poorly diverged subset of species from euk_db (sub_euk_db). This tree was later used as reference to phylogenetically classify the remaining potential orthologs found in euk_db (supplementary figs. S4–9, Supplementary Material online). 4) Removal of false species-specific subunit paralogs. 5) *TBlastN* (Altschul et al. 1990) and *HMM*-based (Eddy 2011) searches using custom profiles in order to identify previously undetected highly divergent orthologs, which were validated and classified using phylogenetic inference methods. See Supplementary Information Methods, Supplementary Material online for a detailed explanation of the pipeline.

### Inference of Duplication and Losses of ORC Subunits

We first constructed a consensus eukaryotic species tree based on recent bibliographical references (James et al. 2006; Dunn et al. 2014; Ruhfel et al. 2014; Kurtzman et al. 2015; Lowe et al. 2015; Derelle et al. 2016; He et al. 2016; Qiu et al. 2016; Sierra et al. 2016; McCarthy and Fitzpatrick 2017; Munoz-Gomez et al. 2017; Simion et al. 2017; Torruella et al. 2018). We generated one tree per subunit family (ORC2, ORC3, ORC4, and ORC5; CDC6 and ORC1 in the same tree). The sequences included in those trees were the “bona fide” and “likely” subunit members previously identified (false paralogs were excluded), as well as the sequences detected by *TBlastN* and *HMM* searches (see Supplementary Information Methods, Supplementary Material online). Duplications and losses were manually inferred in those ancestral nodes of figure 2 phylogeny in which the inference minimizes the number of events required to explain the distribution of ORC subunits in euk_db, while being compatible with the phylogenetic signal. For example, losses of ORC2-5 were inferred in the root of *Entamoeba* as none of the three *Entamoeba* species in euk_db has an ORC2-5 sequence (see Ehis, Enut, and Einv in fig. 2). However, in the case of Blastocladiomycota (Fungi), despite the two species from this group (Cang and Amac) have two ORC4 copies, we inferred two independent duplications in Cang and Amac instead of a single duplication in their last common ancestor because the phylogeny indicate that they are species-specific paralogs (supplementary fig. 7, Supplementary Material online).

### Analyses of PACT Domain Region Conservation

All bona fide ORC1 sequences were separately aligned to the *H. sapiens* sequence [*MAFFT*: mafft-einsi]. Alignments were split in two subalignments, one including the positions corresponding to the two motifs of the PACT region of *H. sapiens* ORC1 (Hossain and Stillman 2012) (PACT), and another including the positions outside the motifs (non-PACT). Identity and similarity measures for each subalignment were obtained using the *myseqs* function (*seqinr* R package). The Fitch matrix (Fitch 1966) was used for similarity measures. Identity and similarity measures of PACT subalignments were divided by the measures of the corresponding non-PACT subalignment. For those taxa with presence/absence of centrioles information available (Carvalho-Santos et al. 2011), the resulting values were classified in two categories: “Centrioles” and “No centrioles.” We used the *boxplot* and the *wilcox.test* R functions to represent and compare the identity and similarity distributions between the two sets, respectively.

### *Chlamydomonas reinhardtii* Culture and Generation of amiRNA ORC1

*Chlamydomonas reinhardtii* cells were cultured in Tris-acetate-phosphate (TAP) containing 8 mM ammonium chloride or 25 mM potassium nitrate, as indicated, at 25 °C under continuous light and agitation, till exponential growth phase. Artificial miRNA lines against *C. reinhardtii* ORC1 (Cre10.g455600.t1.1) were generated (Molnar et al. 2009) using the scaffold pChlamyNR-RNA3 plasmid, under the control of the NITRATE REDUCTASE (NR) promoter, using primers listed in supplementary table 2, Supplementary Material online. Transformants were generated as described (Kindle 1990; Loppes et al. 1999) and selected in TAP medium containing ammonium as nitrogen source and supplemented with 25 μg/ml paromomycin. ORC1 transcript levels were measured by quantitative-PCR using primers indicated in supplementary table 2, Supplementary Material online and the iScript cDNA synthesis kit (Bio-Rad) and normalizing RNA levels to the ubiquitin ligase gene (Gonzalez-Ballester et al. 2004).

### Motility and Immunocytochemical Assays

Motility was analyzed under optical microscope (Leica DM750) as the number of cells crossing a square of a Neubauer chamber during 10 s. For immunocytochemical identification of centrioles and flagella, cells were recovered, adjusted to 10^7^ cells/ml, placed on a poly-l-lysine-coated slide, incubated for 10 min at room temperature and fixed with 4% paraformaldehyde. Immunolocalization was performed as described (Uniacke et al. 2011), using anti-acetylated tubulin antibody (Clone 6-11B-1, Sigma, dilution 1:1000) and daylight 488 horse antimouse antibody (Vector Laboratories DI-2488, 1:500). Nuclei were counterstained with DAPI.

## Results

### Phylogenetic Relationships between ORC Subunits

**Fig. 1.**
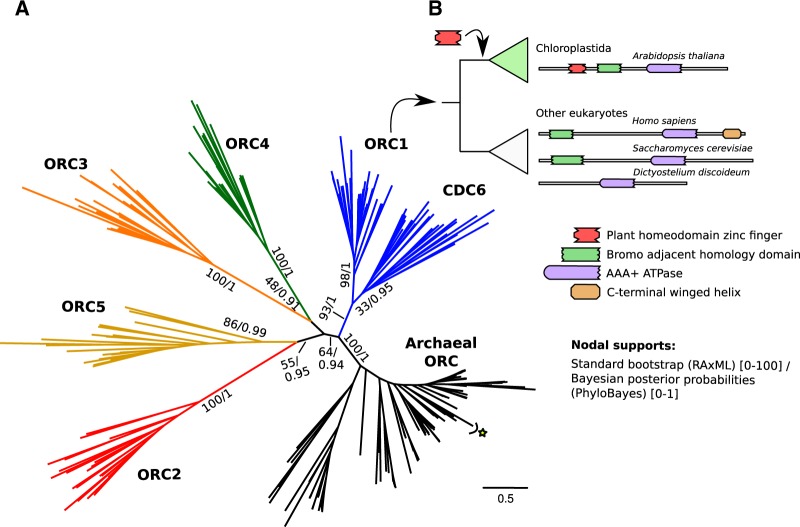
—(*A*) Maximum likelihood tree (RAxML) including the CDC6 and ORC1-5 subunits from a subsampling of eukaryotic sequences (sub_euk_db) as well as archaeal sequences selected for rooting purposes. The position of Asgard archaea sequences is indicated by a star symbol (see supplementary fig. 3, Supplementary Material online for the complete representation of the tree). Two nodal support metrics are represented: 1) standard nonparametric bootstraps computed using RAxML software (“PROTGAMMALG” model, 100 bootstrap replicates). 2) Bayesian posterior probabilities computed with PhyloBayes software (“LG + gamma4” model, see the consensus Bayesian tree in supplementary fig. 12, Supplementary Material online). (*B*) Representation of the PHD domain insertion occurred in the ORC1 gene of a common ancestor of land plants and green algae (Chloroplastida). Chloroplastida ORC1 thus shows a distinct domain architecture with respect to other eukaryotes. See supplementary fig. 1, Supplementary Material online for a representation of the domain architectures found for all bona fide ORC1 euk_db sequences.

Our phylogenetic tree (fig. 1*A*, see extended version in supplementary fig. 3, Supplementary Material online), constructed from a subsampling of taxa (sub_euk_db, see Materials and methods), shows all eukaryotic subunits (i.e., CDC6 and ORC1–ORC5) branching in a separate clade than the archaeal homologs (100% nodal support) (fig. 1*A*). This suggests that all eukaryotic subunits (CDC6+ORC1-5) originated from a single archaeal paralog and not from distinct paralogs as previously suggested (Makarova and Koonin 2013), with a first duplication leading to pre-CDC6/ORC1 and to pre-ORC2-5 paralogs. The subunits from ORC2 to ORC5 would have originated from subsequent duplications of the second paralog. However, the duplication order is uncertain (low nodal supports, see fig. 1*A*), which is probably a consequence of the high divergence levels between and within ORC subunits. Divergence within subunits is also observed in protein domain architecture. In ORC1, *AAA+ ATPase* is the only domain conserved (supplementary fig. 1, Supplementary Material online), whereas the *Bromo adjacent homology domain* (BAH) and *C-terminal winged helix* (Cdc6_C) are both patchily distributed, the last only found in Holozoa (Metazoa + relatives) and Holomycota (Fungi + relatives). Because these two domains have been shown to be essential for cell cycle progression (Kuo et al. 2012) and the stability of ORC in Metazoa (Bleichert et al. 2015), its absence from some ORC1 may well be because of a lack of sensitivity of the corresponding HMM profiles from Pfam database (Finn et al. 2014). The *Plant homeodomain zinc finger domain* (PHD), described in *Arabidopsis* ORC1 as being involved in chromatin interaction functions (Sanchez and Gutierrez 2009; Sanchez and Zhou 2011; Li et al. 2016), is also found in other ORC1 sequences from Chloroplastida (land plants + green algae) (supplementary fig. 1, Supplementary Material online). The PHD region from Chloroplastida ORC1 only shows similarity to non-ORC eukaryotic proteins (supplementary fig. 2, Supplementary Material online, see supplementary methods, Supplementary Material online), suggesting that it was incorporated into ORC1 through a domain rearrangement with a PHD-bearing protein in the root of Chloroplastida.

The “Archaeal ORC” clade in figure 1*A* includes those noneukaryotic sequences that aligned with the highest score to the eukaryotic CDC6+ORC1-5 sequences. Within this clade, sequences from Asgard archaea group (see star symbols in fig. 1*A* and supplementary fig. 3, Supplementary Material online) branch in a distant position with respect to eukaryotes. This contrasts with the fact that Asgards have been proposed by some authors to be the closest archaeal lineages to eukaryotes (Zaremba-Niedzwiedzka et al. 2017). Many factors may explain this unexpected topology. On the one hand, there is still some controversy with regards the relation of Asgard lineages and eukaryotes (Da Cunha et al. 2018; Spang et al. 2018). On the other hand, even if they are the sister-group to eukaryotes, their distant position to eukaryotes in the ORC phylogeny (fig. 1*A*) may be explained either by 1) methodological limitations during the phylogenetic inference; by 2) a convoluted evolutionary scenario involving, for example, an HGT-acquisition of ORC in the stem lineage of eukaryotes by a non-Asgard archaeal lineage; or by 3) ancestral subunit paralogs that may had been differentially lost in Asgards and in eukaryotes. Notwithstanding this remaining uncertainty, the recovered topology is clear with the fact that the duplications leading to the eukaryotic ORC paralogs (CDC6 and ORC1-5) would have occurred in eukaryotes after the divergence from all currently known Archaea. Still, the specific eukaryotic lineage in which ORC would have completely diversified remains uncertain because of the finding of highly divergent subunit homologs in Metamonada and Discoba taxa and also because of the uncertain phylogenetic position of these two groups in the eukaryotic species tree (see Discussion or the “Origin of the eukaryotic ORC” Supplementary Information Results section, Supplementary Material online for a detailed explanation of the potential scenarios for ORC origins in eukaryotes).

### Evolutionary Dynamics of ORC in Eukaryotes

**Fig. 2.**
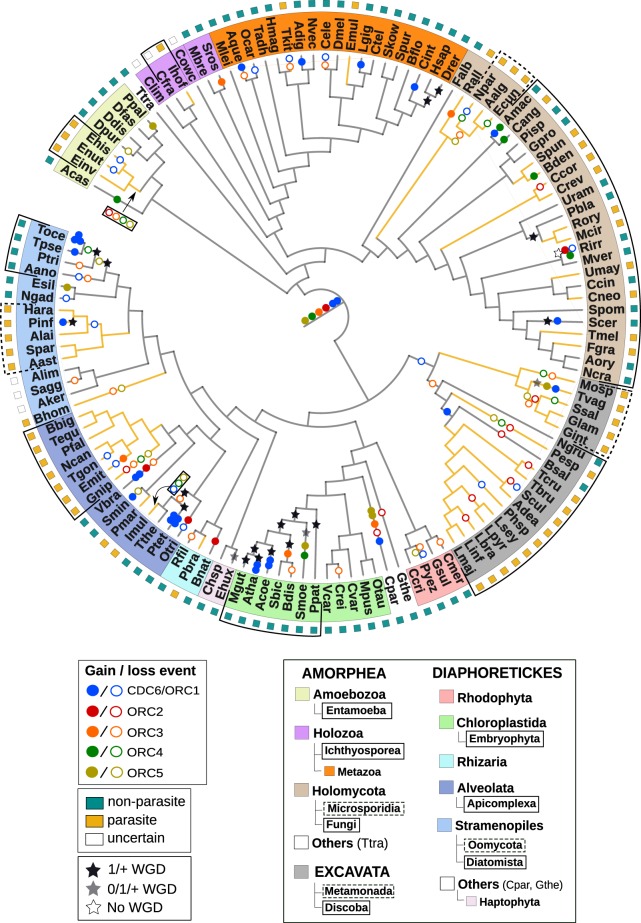
—Evolutionary history of ORC in eukaryotes. Duplications and losses of ORC subunits are mapped in a consensus eukaryotic species tree. The duplications shown in the root represent the origin of CDC6 and ORC1-5 subunits from a single archaeal sequence before the divergence of all sampled eukaryotes (other scenarios are possible, see supplementary fig. 10, Supplementary Material online). For the sake of simplicity, species names are represented in a four-letter code (see supplementary table 1, Supplementary Material online for the correspondence between four-letter and full species names, as well as for information regarding the number of ORC subunit copies per species). Branches corresponding to parasitic lineages are colored in yellow. Parasitic lifestyle was inferred for all ancestral lineages from which all descendant species are parasites. Information of whole-genome duplications (WGD) reported in the bibliography is also highlighted. “1/+ WGD”: at least 1 WGD would have occurred; “0/1/+1 WGD”: WGD reported only by some references; “No WGD”: WGD would not have occurred.

**Fig. 3.**
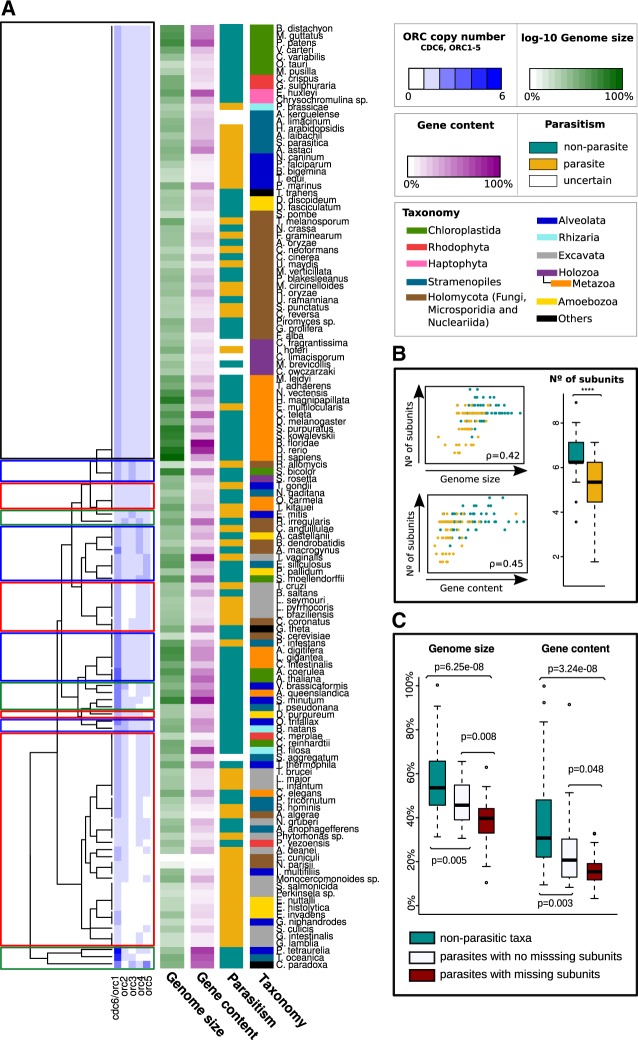
—(*A*) Clustering of eukaryotic taxa according to ORC subunits counts (see Supplementary Information Methods, Supplementary Material online). In the heatmaps, Genome size (GS) metrics were normalized to the largest genome in euk_db after being converted into base-10 log scale. Gene content (GC) metrics were also normalized to the largest number of sequences in euk_db. (*B*) On the left, scatter plots illustrating the correlation between total CDC6+ORC1-5 counts and genome size and gene content metrics. Dots are colored according to species lifestyle (parasite or nonparasite). Spearman’s correlation coefficients (*ρ*) are also represented. On the right, the distribution of total CDC6+ORC1-5 counts in parasitic and nonparasitic taxa (Mann–Whitney *U* test *P*-value = 1.165e–05). (*C*) Distribution of genome size and gene content metrics for nonparasitic taxa, parasites with no missing ORC subunits, and parasites with missing subunits. Mann–Whitney *U* test *P*-values (*P*) are indicated.

Based on ORC phylogenies (supplementary figs. 4–9, Supplementary Material online), we parsimoniously inferred duplication and loss events in order to explain the number of ORC subunits found in extant taxa (fig. 2*A*). For this purpose, CDC6 and ORC1 were considered as the same ORC subunit (CDC6/ORC1) given the existence of uncertain CDC6/ORC1 sequences (supplementary fig. 9, Supplementary Material online). The eukaryotic species tree was rooted between Amorphea and Diaphoretickes + Excavata (Derelle et al. 2015; Betts et al. 2018). According to this root, the LECA would have had 2 CDC6/ORC1 copies (CDC6 and ORC1) and 1 copy of ORC2-5 subunits (see supplementary fig. 10*B*, Supplementary Material online and Supplementary Information Results, Supplementary Material online for distinct LECA ORC subunit configurations according to alternative roots). This ancestral ORC configuration consisting in 6 subunits is by far the most represented in our data set (49/132 taxa). The mean number of ORC subunits is 5.51, which could be interpreted as only a minor tendency of eukaryotes to simplify its subunit configuration along evolution. However, we found substantial differences between taxa, ranging from 1 ORC sequence in *Spironucleus salmonicida* (Metamonada) to 9 in *Paramecium tetraurelia* (Ciliophora, Alveolata) (fig. 3*A*, supplementary table 1, Supplementary Material online). Remarkable differences are also observed between phylogenetically related species (fig. 2*A*). For example, in ciliates, we found five subunits in *Tetrahymena thermophila* but only one in the parasite *Ichthyophthirius multifiliis*. In total, we inferred 69 loss and 47 duplication events involving all CDC6+ORC1-5 subunits (fig. 2*A*). In particular, 18, 12, 19, 9, and 11 losses and 26, 4, 4, 6, and 6 duplications for CDC6/ORC1, ORC2, ORC3, ORC4, and ORC5, respectively. ORC3 appears as the less conserved subunit, followed by ORC5, ORC2, ORC4 (absent in 38, 19, 17, and 14 taxa, respectively). While we found CDC6/ORC1 subunits in all taxa, 20 conserve only a single copy, indicating that eukaryotes evolved alternative ORC configurations in which the presence of both CDC6 and ORC1 is not essential. The finding of losses involving all subunits agrees with no one being strictly indispensable globally in eukaryotic evolution (Aves et al. 2012). Accordingly, in vitro loss-of-function mutations in ORC1 and ORC2 are not lethal in human cells (Shibata et al. 2016), possibly reflecting the intrinsic potential of eukaryotes to evolve alternative ORC configurations.

Previous works described ORC as a conserved eukaryotic feature (Duncker et al. 2009). Overall, our results only partially agree with this designation. The dendrogram in figure 3*A* (see Supplementary Information Methods, Supplementary Material online) shows a clustering of species according to their ORC subunit counts. From the top down, a first major cluster (black square) includes all taxa with the ancestral ORC configuration (1 copy of every subunit, CDC6 and ORC1 counted together). Taxa from all eukaryotic supergroups except Excavata are represented in this cluster. The rest of the dendrogram includes taxa with at least one extra paralog (blue squares), taxa with at least one subunit absent (red squares), and taxa that have extra paralogs but lost some subunits (green squares). Overall, despite the fact that the conservation of the ancestral subunit configuration generally seems to be favored, many lineages from distinct parts of the eukaryotic tree explored alternative subunit configurations. Rather than being an exclusive feature of eukaryotes, the number of ORC subunits is also variable in Archaea, ranging from 1 to 20 paralogs. The acquisition of subunit paralogs was proposed to be related with the appearance of additional origins of replication (ORIs) in archaeal genomes (Makarova and Koonin 2013).

In eukaryotes, ORIs have only been quantified in some model organisms, and seems to vary between species and even between cell-types (Leonard and Mechali 2013; Pourkarimi et al. 2016; Rodriguez-Martinez et al. 2017; Sequeira-Mendes et al. 2019). Still, the data available so far suggest that the number of ORIs would be proportional to genome size (Spearman correlation coefficient *ρ* = 0.79; supplementary fig. 11, Supplementary Material online). Despite us finding a certain correlation between genome size and number of ORC subunits (fig. 3*B*), ORC evolution is unlikely to be directly influenced by increments of ORIs, as for example *Saccharomyces* does not have less subunits than *Homo* despite its genome being ∼250 times smaller (fig. 3*A*). Therefore, whereas expression levels of ORC subunits are a limiting factor for ORIs activity (Wong et al. 2011), the number of ORC subunit paralogs is unlikely to be a constraint for the acquisition of novel ORIs. We thus propose that the observed correlation between the number of ORC subunits and genome size is because this is highly correlated with gene content (*ρ* = 0.79). In particular, we propose that global changes in gene content, promoted for example by WGD or streamlining evolution (Giovannoni et al. 2014) may lead to changes in the number of subunit paralogs (*ρ* = 0.45 correlation between number of ORC subunits and gene content, fig. 3*B*).

The distribution of losses and gains of subunits also agrees with the influence of global gene turnover rates on ORC evolution (fig. 2*A*). On the one hand, we found subunit losses to be enriched in parasitic lineages (41/69 losses in the 83/263 parasitic lineages of fig. 2*A*, one-tailed Fisher’s Exact Test *P*-value = 2.43e–05). Consequently, the number of subunits is significantly lower in parasites (fig. 3*B*). However, not all parasitic lineages reduced the number of ORC subunits. 37/41 losses in parasites occurred specifically in Excavata, Apicomplexa, Microsporidia, Entamoeba, *Blastocystis hominis*, and *I. hoferi*. While both parasites with and without missing subunits show significantly lower genome size and gene content metrics than nonparasitic taxa, differences are greater in parasites with missing subunits (fig. 3*C*). This suggests that convergent losses of subunits occurred in these lineages because the selective pressure favoring genome streamlining overcame the constraints promoting the conservation of the complex (Corradi and Slamovits 2011; Coyne et al. 2011; Giovannoni et al. 2014; Janouskovec et al. 2015; Jackson et al. 2016). On the other hand, lineages in which WGD have been reported are enriched in ORC subunit duplications (11/39 duplications in the 20/263 WGD-lineages of fig. 2*A*, one-tailed Fisher’s Exact Test *P*-value = 2.117e–05) (Aury et al. 2006; Carlton et al. 2007; Van de Peer et al. 2009; Panchy et al. 2016; Corrochano et al. 2016; Carrier et al. 2017; Clark and Donoghue 2018; Parks et al. 2018; Yang et al. 2018; Morin et al. 2019; Qiao et al. 2019). In some cases, WGDs were also accompanied by losses of subunits (four losses in WGD-lineages). For example, *P. tetraurelia* has six CDC6/ORC1 paralogs, possibly retained after the multiple rounds of genome duplication occurred in this ciliate lineage (Aury et al. 2006). In contrast, paralogs of ORC2, ORC4, and ORC5 would not have been retained, and ORC3 was lost in the common ancestor shared with *T.* *thermophila* and *I. multifiliis* (fig. 2*A*).

### Origin of ORC1 Role in Controlling Centriole Duplication

**Fig. 4.**
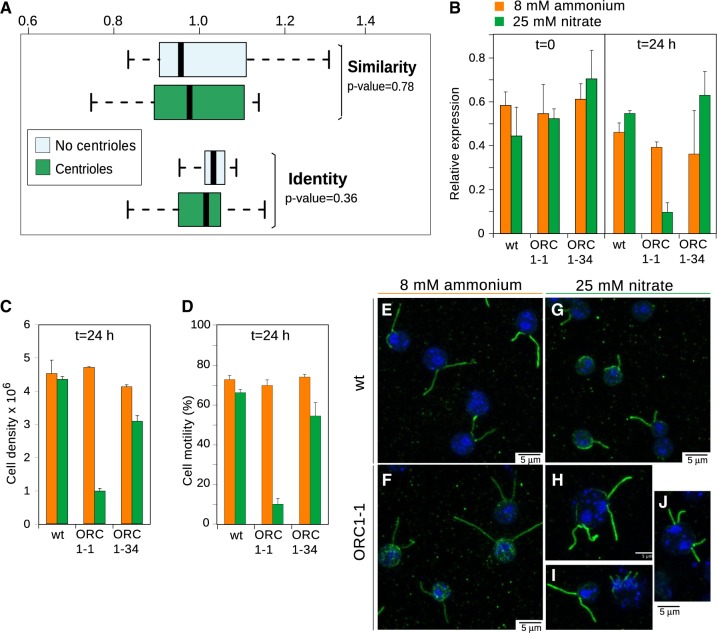
—(*A*) Degree of conservation of *Homo sapiens* ORC1 PACT region with respect to the rest of the protein in taxa with and without centrioles (see Materials and methods). Differences in the distributions were tested with the Mann–Whitney *U* test (see *P*-values in the figure). (*B*) Relative expression of CrORC1 was determined in wt and transformants (ORC1-1 and ORC1-34) for the amiRNA against CrORC1 in the absence (8 mM ammonium; orange) or presence (25 mM nitrate; green) of amiRNA-inducing conditions. CrORC1 mRNA levels were determined before (*t* = 0) of 24 h after changing the medium to inducing conditions (*t* = 24). (*C*) Cell density was measured 24 h after changing exponential cultures grown in ammonium (orange) to the amiRNA-inducing medium containing nitrate (green). (*D*) Same as in panel B but quantifying cell motility. (*E*–*J*) Images of wt (*E*, *G*) and ORC1-1 strain expressing amiRNA against CrORC1 (*F*, *H*–*J*) *Chlamydomonas reinhardtii* cells grown in noninducing (8 mM ammonium; *E*, *F*) or inducing (25 mM nitrate; *G*–*J*) medium. Centrioles and flagella were visualized by immunostaining with antitubulin b (green). Nuclei were counterstained with DAPI (blue).

Functional implications of recent subunit duplications include examples of both subfunctionalization and neofunctionalization. *Arabidopsis* has two ORC1 paralogs, AtORC1a and AtORC1b, highly conserved at amino acid level but differentially expressed in distinct cell-types (Diaz-Trivino 2005). In contrast, the ORC1 paralog of *S. cerevisiae* (Sir3) is not involved in ORC but in heterochromatin formation (Bell et al. 1995). Still, it remains unclear if the great increment of subunits occurred early in eukaryotes (from an archaeal-like CDC6/ORC1 to CDC6+ORC1-5) involved only a subfunctionalization process or was also accompanied by the neofunctionalization of some subunits. Despite alternative functions besides ORI recognition have been described in eukaryotes with the canonical ORC configuration (Chesnokov 2007; Ortega et al. 2016), it is uncertain whether these are ancestral or lineage-specific acquisitions. For example, ORC1 was found to be involved in controlling centriole duplication in humans and mouse (Hemerly et al. 2009; Hossain and Stillman 2012). In *Homo*, the region involved in targeting HsORC1 to the centriole consists in two small motifs located in the C-terminus of HsORC1 (PACT region). Although centrioles are absent in many lineages (Carvalho-Santos et al. 2011), their presence in fairly unrelated eukaryotes indicate that they originated in an ancestral lineage, as ORC1. However, it is unclear whether the role of HsORC1 in centrioles is also ancestral or was recently acquired in a lineage leading to *Homo*. We checked for bioinformatics evidences of the presence of this function in other eukaryotes with centrioles. If the role in centrioles through the PACT region is ancestral and conserved in eukaryotes and this region is specifically involved in this function, we would expect the alignment positions corresponding to PACT in HsORC1 to be conserved only in centriole-bearing eukaryotes. Our results reject this hypothesis, as in average the PACT region is not significantly more conserved in centriole-bearing eukaryotes (fig. 4*A*).

We envisioned two plausible scenarios. Either the role in centrioles was independently acquired in the lineage leading to animals, or the role was ancestral but the protein region mediating it would have diverged. We addressed these hypotheses in the unicellular biflagellated green alga *C.* *reinhardtii*, a member of the Chlorophyta group, which is distantly related to animals in the eukaryotic tree (fig. 2*A*). We used amiRNA silencing of CrORC1 to determine whether the phenotype observed is related to centriole homeostasis. We transformed the wild type algal strain 704 (wt) with an amiRNA-nitrate inducible-expressing plasmid, paromomycin-resistant transformants were selected and, then, we screened for those showing deficient growth under selection conditions. Two transformants, ORC1-1 and ORC1-34, showed strong and mild growth deficiency, respectively, in nitrate medium. CrORC1 mRNA levels in cells grown in normal medium until exponential phase (*t* = 0) were normal whereas after transferring them (at 10^6^ cells/ml) to a medium inducing the amiRNA against CrORC1 (*t* = 24). The ORC1-1 strain showed a ∼80% reduction in CrORC1 mRNA levels (fig. 4*B*). Consistent with the reduced CrORC1 expression, we observed a severe growth reduction of the ORC1-1 strain in the presence of nitrate but not with other strains (fig. 4*C*). Likewise, reduction of CrORC1 levels led to a significant defect in motility of the ORC1-1 strain (fig. 4*D*), revealing that ORC1 is required for proper cell growth and motility of algal cells.

To test if the motility defects were associated with defects in MTOC homeostasis, we visualized centrioles and flagella by immunofluorescence using antitubulin—antibodies. Both wild type and ORC1-1 cells showed a normal appearance in the absence of amiRNA production (fig. 4*E* and *F*). In contrast, we found an abnormal phenotype in the ORC1-1 cells grown in nitrate (fig. 4*G*–*J*). The defective cells contained two enlarged nuclei although the proportion of centrioles to flagella and nuclei was not affected, in contrast with the increased number of centrioles in human cells after knockdown of ORC1 (Hossain and Stillman 2012). Therefore, we found that reduced levels of CrORC1 led to division defects but not to an unrestricted number of centrioles. These results are consistent with the idea that ORC1 does not regulate centriole homeostasis in *Chlamydomonas*.

## Discussion

Our phylogenetic reconstruction of the evolutionary history of the CDC6 and ORC1-5 subunits showed that the evolution of this protein complex is not as simple as previously proposed (Duncker et al. 2009). Taking into account the inferred phylogeny (fig. 1) and the subunit distribution across eukaryotes (fig. 3), we determined that CDC6 and ORC1 to ORC5 diversified from a single ancestral archaeal-like orc1/cdc6 gene. This occurred after eukaryotes diverged from Asgard archaea and before the divergence of Diaphoretickes (plants, algae, and others) and Amorphea (animals, fungi, and others). Difficulties to pinpoint a more precise origin lays on the uncertain position of Metamonada (*Giardia*, *Trichomonas*, and others) and Discoba (Trypanosomatida and others) in the eukaryotic tree (Adl et al. 2019), and also on the distribution of ORC subunits in these two groups (supplementary fig. 10*A*, Supplementary Material online) (see “Origin of the eukaryotic ORC” Supplementary Information Results section, Supplementary Material online for a detailed explanation of the potential scenarios for ORC origins in eukaryotes). Still, the most likely scenario is that a completely diversified ORC would have been already present in the last eukaryotic common ancestor (LECA). This scenario would certainly be true if neither Metamonada nor Discoba originated earlier than Amorphea and Diaphoretickes (see H1 in supplementary fig. 10*B*, Supplementary Material online). But even in the opposite scenario, the pre-LECA origin of CDC6+ORC1-5 is also more parsimonious given the found distribution of subunit orthologs in Metamonada and Discoba (supplementary fig. 10*A*, Supplementary Material online). In particular, despite most taxa from both groups showing reduced ORC subunit configurations, the last common ancestor of Metamonada and of Discoba probably had a completely diversified ORC (see H2A–H4A in supplementary fig. 10*B*, Supplementary Material online), as putative orthologs of all the subunits are found in at least one taxa of both groups (except ORC3 in Discoba). Still, we cannot rule out that these sequences, because of being highly diverged, may correspond to prediversified ORC subunits (see H2B–H4B in supplementary fig. 10*B*, Supplementary Material online). Future genome sampling efforts of nonparasitic taxa from Metamonada and Discoba as well as from relative lineages could possibly help to solve these remaining uncertainties.

Despite one copy of every ORC subunit being the most represented configuration in eukaryotes (fig. 3*A*), >60% of taxa show variations because of loss and duplication events. We found that variations in the number of subunits respond to the tendency of a genome to either increase or decrease its genetic content (fig. 3). In particular, >50% of losses were found specifically in parasitic groups with streamlined genomes as shown by their lower genome size and gene content metrics (fig. 3*C*) (Corradi and Slamovits 2011; Coyne et al. 2011; Giovannoni et al. 2014; Janouskovec et al. 2015; Jackson et al. 2016). WGD also appear to be behind the acquisition of paralog subunits (fig. 2). Still, not all WGD events involved changes that can be observed in extant taxa, as for example *Homo* conserve a single copy of every subunit despite the WGDs occurred in the vertebrate lineage (fig. 2) (Van de Peer et al. 2009). Importantly, gene duplications of ORC subunits do not necessarily imply changes in the complex. Instead of becoming a novel members of the complex, the acquired paralogs may evolve alternative functions such as the *S. cerevisiae* ORC1 paralog Sir1, which acts as a transcriptional repressor by binding to ORC1 (Bell et al. 1995). Indeed, most of the subunit paralogs found appear to be recent acquisitions (fig. 2), which suggest a tendency of paralogs to be lost along evolution. This could be explained because, in general, ORC subunit paralogs may evolve functions that do not become essential enough to be retained. However, in some lineages, the duplications have been accompanied or occurred in parallel to losses of other subunits (fig. 2). An experimental determination of the ORC in these species would clarify whether the duplicated subunits had replaced canonical subunits in the complex.

It is also important to stress that the inference of subunit losses is not a direct proof of reduced ORCs. *Trypanosoma* *brucei* is, to the best of our knowledge, the only species in which a putatively reduced ORC has been characterized (Marques et al. 2016). Whereas initially an ORC1/CDC6 protein was thought to be the only subunit as in Archaea (Godoy et al. 2009), three further members of the complex were experimentally characterized (Marques et al. 2016). One of these members was proposed to be a remote ORC4 ortholog, and the other two were proposed to be putative orthologs of ORC2 and ORC5 based on similarities only detected at structural level. Our sequence-similarity based orthology detection pipeline not only detected but also extended the presence of these subunits to other Discoba and Metamonada, and also confirmed the identity of ORC2 and ORC5 (fig. 3, supplementary fig. 10*A*, Supplementary Material online). This suggests a good sensitivity for our detection pipeline, and hence that taxa in which subunit losses were inferred (fig. 2) are likely to bona fide lack these ORC components. The experimental characterization of ORC in lineages with rampant losses such as *Entamoeba* (fig. 2) would confirm whether these lineages reversed their ORC into an archaeal-like configuration with just one subunit, or whether the canonical subunits have been replaced by alternative protein components. Further experimental analyses are also required to determine the origin of neofunctionalizations described for some subunits such as the role of ORC1 in controlling the duplication of centrioles in human cells (Hemerly et al. 2009). Our knockdown experiments of ORC1 in the green alga *C. reinhardtii* did not lead to increments in the number of centrioles per nuclei (fig. 4*B*–*J*), in contrast to what occurs in human cells (Hemerly et al. 2009; Hossain and Stillman 2012). This suggests that the control of centriole homeostasis in *C. reinhardtii* is independent of ORC1. Although this is the most likely scenario, other possibilities may occur. Because the cell line used in our experiments is a knockdown of ORC1, it is formally possible that even the highly reduced amount of ORC1 mRNA is still able to produce sufficient protein to achieve a normal centriole regulation. The existence of redundant activities from other proteins is also a possibility. However, both those are unlikely because ORC1 mRNA and protein amount in other systems are rather low and the full knockout of ORC1 is lethal. Another important finding is that the region responsible for the role of ORC1 in centriole homeostasis in humans is not more significantly conserved in eukaryotes with centrioles than in eukaryotes without centrioles (fig. 4). Thus, we can take this observation as a suggestion that this neofunctionalization is not ancestral in eukaryotes but may represent a more recent acquisition.

## Supplementary Material

evaa011_Supplementary_Data

## Notes

**Data deposition:** This project has been deposited at Figshare under the accession https://doi.org/10.6084/m9.figshare.7993256.v1.
