# Supplementary material for: Origin Recognition Complex (ORC) Evolution Is Influenced by Global Gene Duplication/Loss Patterns in Eukaryotic Genomes
Source: Genome Biol Evol. 2020 Jan 28;12(2):3878–89. doi: 10.1093/gbe/evaa011 (PMC7058166; doi:10.1093/gbe/evaa011)

Supplementary Figures

Origin recognition complex (ORC) evolution is influenced by global gene duplication / loss patterns in eukaryotic genomes

Eduard Ocaña-Pallarès[1] (0000-0001-6040-4216), Zaida Vergara[2] (0000-0002-8810-1281), Bénédicte Desvoyes[2] (0000-0001-7116-9821), Manuel Tejada-Jimenez[3] (0000-0003-1702-6015), Ainoa Romero-Jurado[3] (0000-0002-0048-2674), Aurora Galvan[3] (0000-0002-7564-2281), Emilio Fernandez[3] (0000-0001-5957-5392), Iñaki Ruiz-Trillo[1,4,5] (0000-0001-6547-5304), Crisanto Gutierrez[2] (0000-0001-8905-8222)

[1] Institut de Biologia Evolutiva (CSIC-Universitat Pompeu Fabra), Passeig Marítim de la Barceloneta 37-49, 08003 Barcelona, Spain

[2] Centro de Biologia Molecular Severo Ochoa, CSIC-UAM, Nicolas Cabrera 1, Cantoblanco, 28049 Madrid, Spain

[3] Departamento de Bioquímica y Biología Molecular, Facultad de Ciencias, Universidad de Córdoba, Campus de Rabanales, 14071 Córdoba, Spain

[4] Departament de Genètica, Microbiologia i Estadística, Universitat de Barcelona, Av. Diagonal, 645, 08028 Barcelona, Spain

[5] ICREA, Passeig Lluís Companys 23, 08010, Barcelona, Spain

+ Correspondence to inaki.ruiz@ibe.upf-csic.es or cgutierrez@cbm.csic.es

Keywords: Origin recognition complex (ORC), DNA replication, eukaryotic evolution, centriole, gene loss, parasitism

Running title: Evolution of origin recognition complex (ORC) in eukaryotes

Supplementary Figure 1. Maximum-likelihood phylogeny including all bona fide and likely ORC1 subunit sequences in euk_db (black branches) as well as some CDC6 sequences used as outgroup. See Supplementary Table 1 for the correspondence between the four-letter code and the species names. Terminal branches were colored according to the taxonomic color code of the corresponding species. Protein domain architectures of sequences are also schematically represented (figure generated with https://itol.embl.de/).

Supplementary Figure 1

ORC1 phylogeny + protein domain architecture

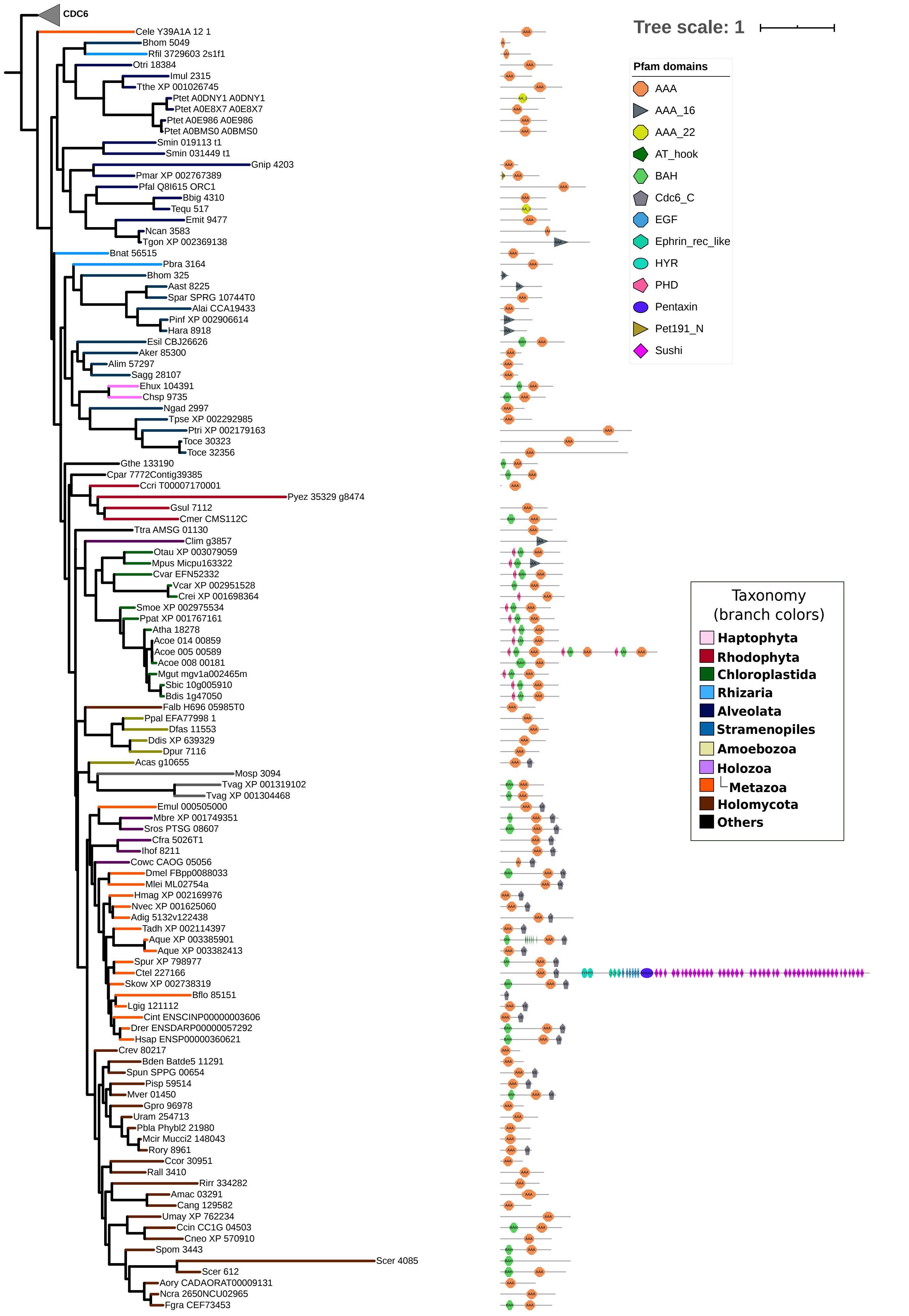

Supplementary Figure 2: Maximum-likelihood phylogenetic tree including all the Chloroplastida ORC1 PHD regions and the captured target sequences from euk_db. Information about the Pfam protein domain architecture is also represented.

Supplementary Figure 2

PHD protein domain phylogeny

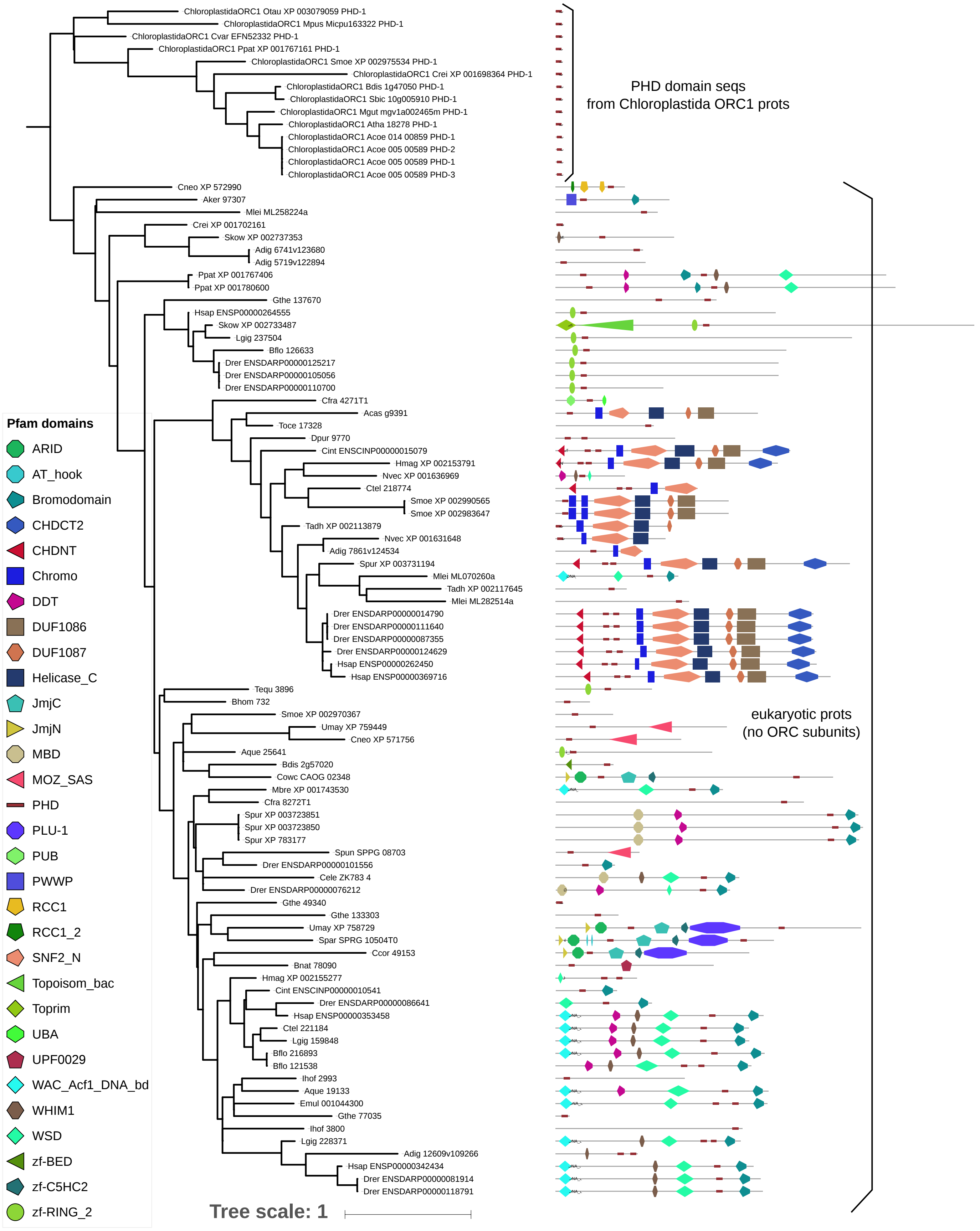

Supplementary Figure 3. Maximum likelihood tree (RAxML) including the CDC6 and ORC1-5 subunits from a subsampling of eukaryotic sequences (sub_euk_db) as well as archaeal sequences selected for rooting purposes. The position of Asgard archaea sequences is indicated by a star symbol. Nodal supports correspond to standard non-parametric bootstraps computed using RAxML software (‘PROTGAMMALG’ model, 100 bootstrap replicates).

Supplementary Figure 3

CDC6+ORC1-5 phylogeny (RAxML)

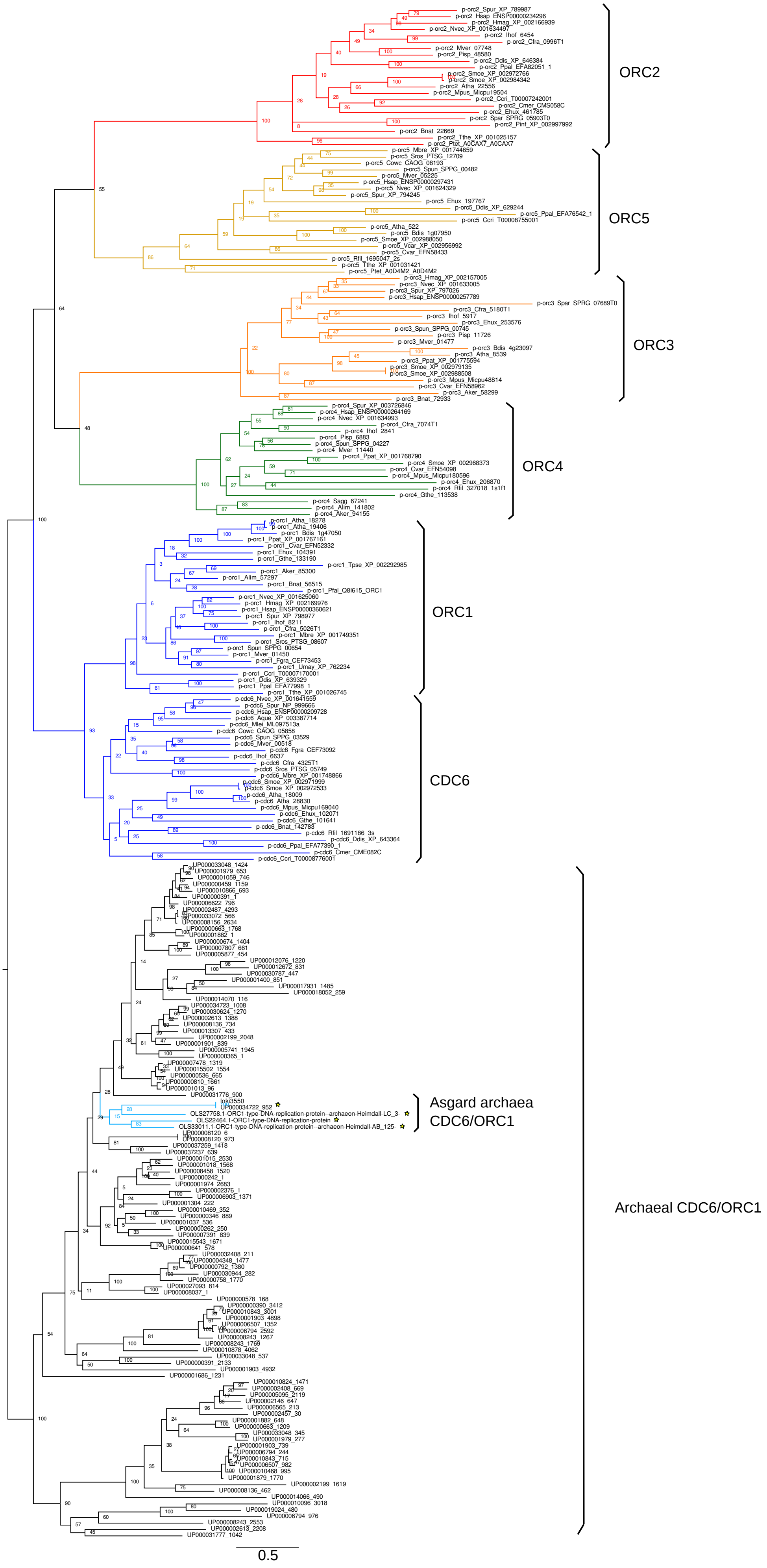

Supplementary Figure 4: Maximum-likelihood phylogenetic tree including all potential CDC6/ORC1 orthologs identified in euk_db as well as the *bona fide* ORC2-ORC5 sub_euk_db sequences identified, used for outgroups purposes. Nodal supports correspond to 100 rapid bootstrap replicates (RAxML). Sequence names start with a prefix indicating the pre-assigned ORC orthogroup using Blast and HMMER-based search approaches (e.g., p-cdc6orc1 corresponds to 'putative CDC6/ORC1') and include the corresponding four-letter code species abbreviature (e.g. Hsap corresponds to *Homo sapiens*. See Supplementary Table 1 for the correspondence between the four-letter code and the species names). Sequence names were coloured according to the taxonomic colour code. CDC6/ORC1 sequences were classified as *bona fide* members (black branches), putative members (orange branches), and unlikely members (red branches). For that, we took into account not only the phylogenetic position in the tree but also additional information such the Pfam protein domain architecture or the results from Blast alignments against other euk_db proteins.

Supplementary Figure 4

Phylogenetic classification of potential CDC6/ORC1 proteins

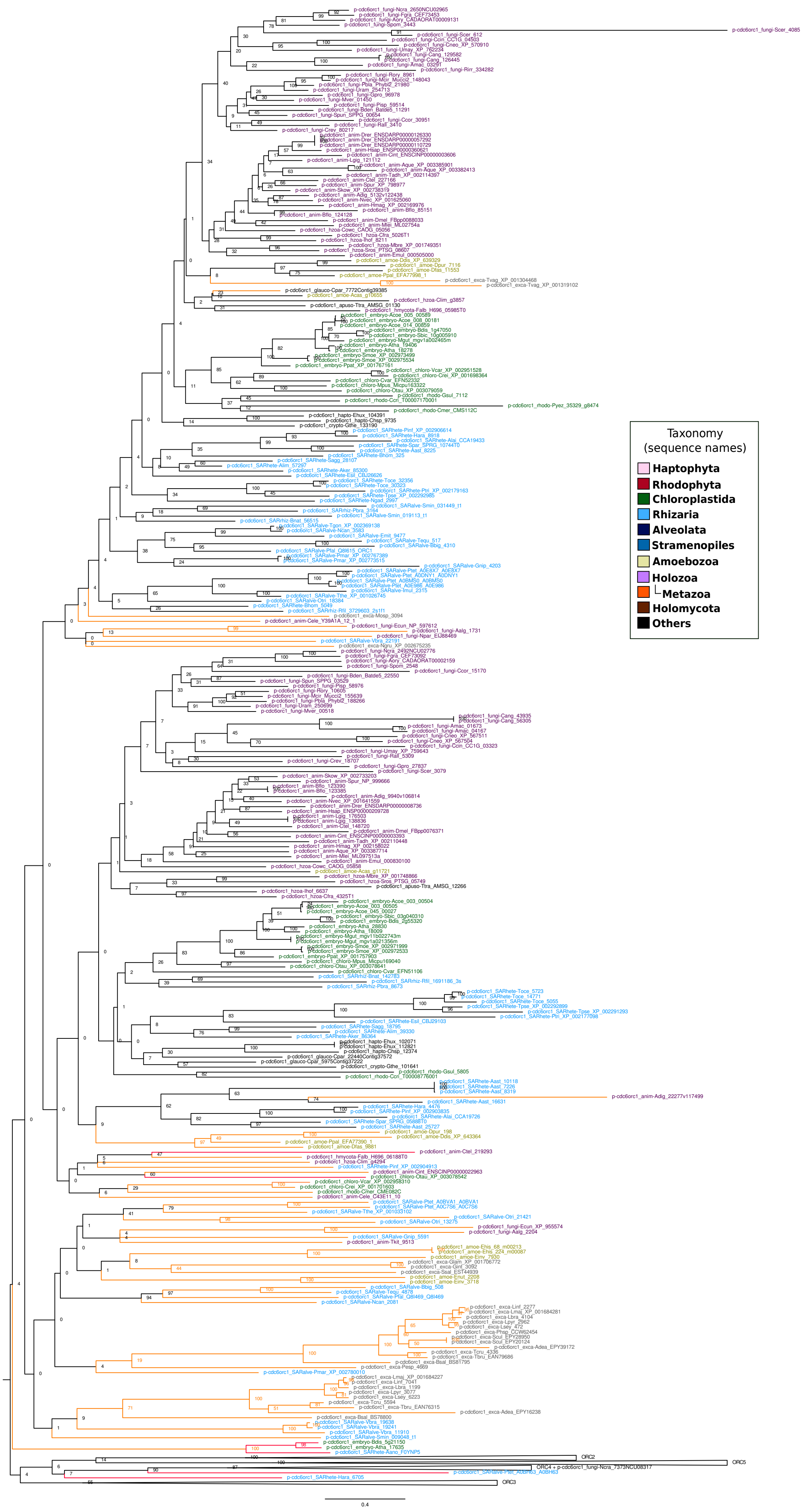

Supplementary Figure 5: Maximum-likelihood phylogenetic tree including all potential ORC2 orthologs identified in euk_db as well as the *bona fide* CDC6/ORC1 and ORC3-ORC5 sub_euk_db sequences identified, used for outgroups purposes. Nodal supports correspond to 100 rapid bootstrap replicates (RAxML). Sequence names start with a prefix indicating the pre-assigned ORC orthogroup using Blast and HMMER-based search approaches (e.g. p-orc2 corresponds to 'putative ORC2') and include the corresponding four-letter code species abbreviature (e.g., Hsap corresponds to *Homo sapiens*. See Supplementary Table 1 for the correspondence between the four-letter code and the species names). Sequence names were coloured according to the taxonomic colour code. ORC2 sequences were classified as *bona fide* members (black branches), putative members (orange branches), and unlikely members (red branches). For that, we took into account not only the phylogenetic position in the tree but also additional information such the Pfam protein domain architecture or the results from Blast alignments against other euk_db proteins.

Supplementary Figure 5

Phylogenetic classification of potential ORC2 proteins

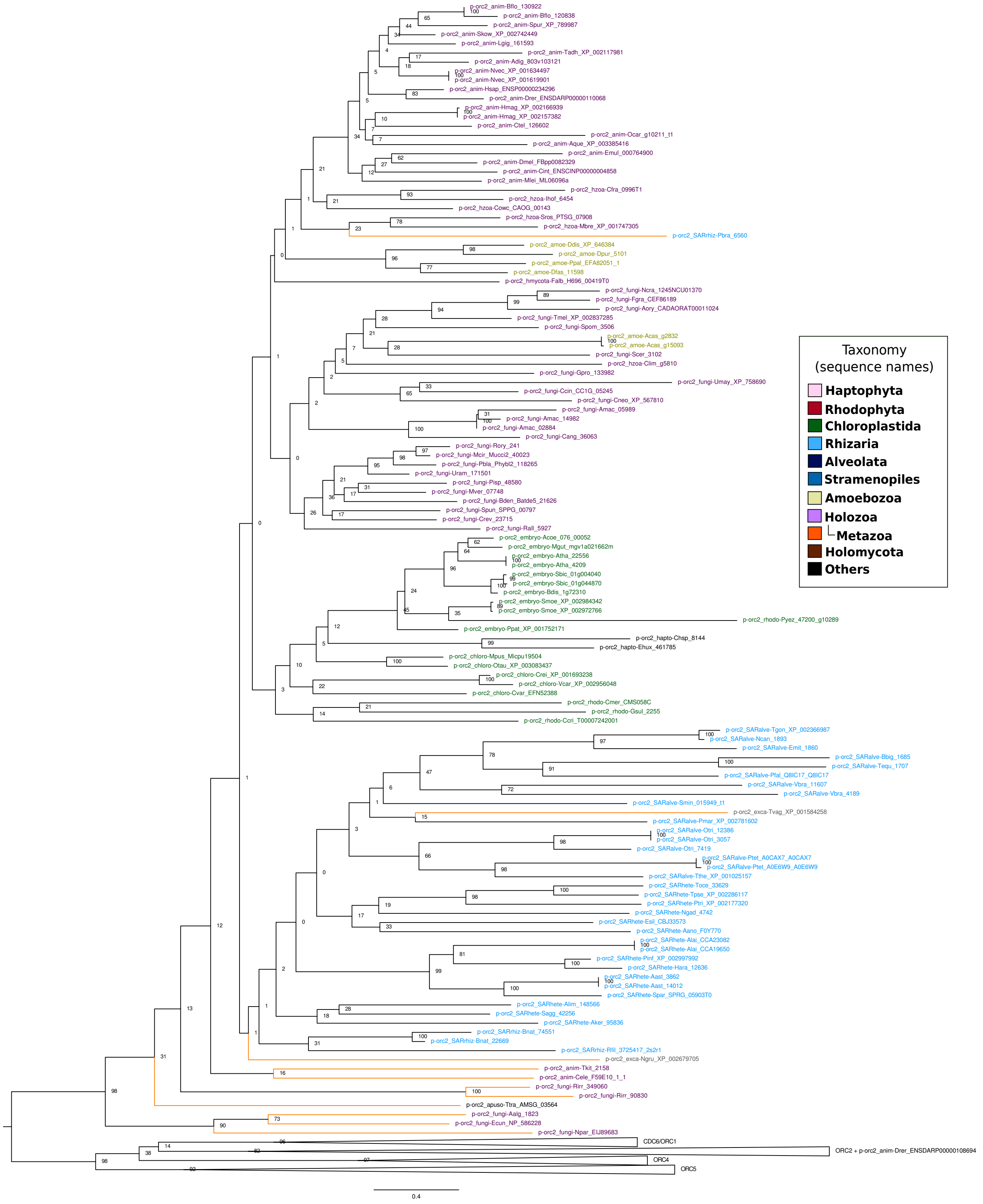

Supplementary Figure 6: Maximum-likelihood phylogeny including all potential ORC3 orthologs identified in euk_db as well as the *bona fide* CDC6/ORC1 and ORC2 and ORC4-ORC5 sub_euk_db sequences identified, used for outgroups purposes. Nodal supports correspond to 100 rapid bootstrap replicates (RAxML). Sequence names start with a prefix indicating the pre-assigned ORC orthogroup using Blast and HMMER-based search approaches (e.g. p-orc3 corresponds to 'putative ORC3') and include the corresponding four-letter code species abbreviature (e.g. Hsap corresponds to *Homo sapiens*. See Supplementary Table 1 for the correspondence between the four-letter code and the species names). Sequence names were colored according to the taxonomic color code. ORC3 sequences were classified as *bona fide* members (black branches), putative members (orange branches), and unlikely members (red branches). For that, we took into account not only the phylogenetic position in the tree but also additional information such the Pfam domain architecture or the results from Blast alignments against other euk_db proteins.

Supplementary Figure 6

Phylogenetic classification of potential ORC3 proteins

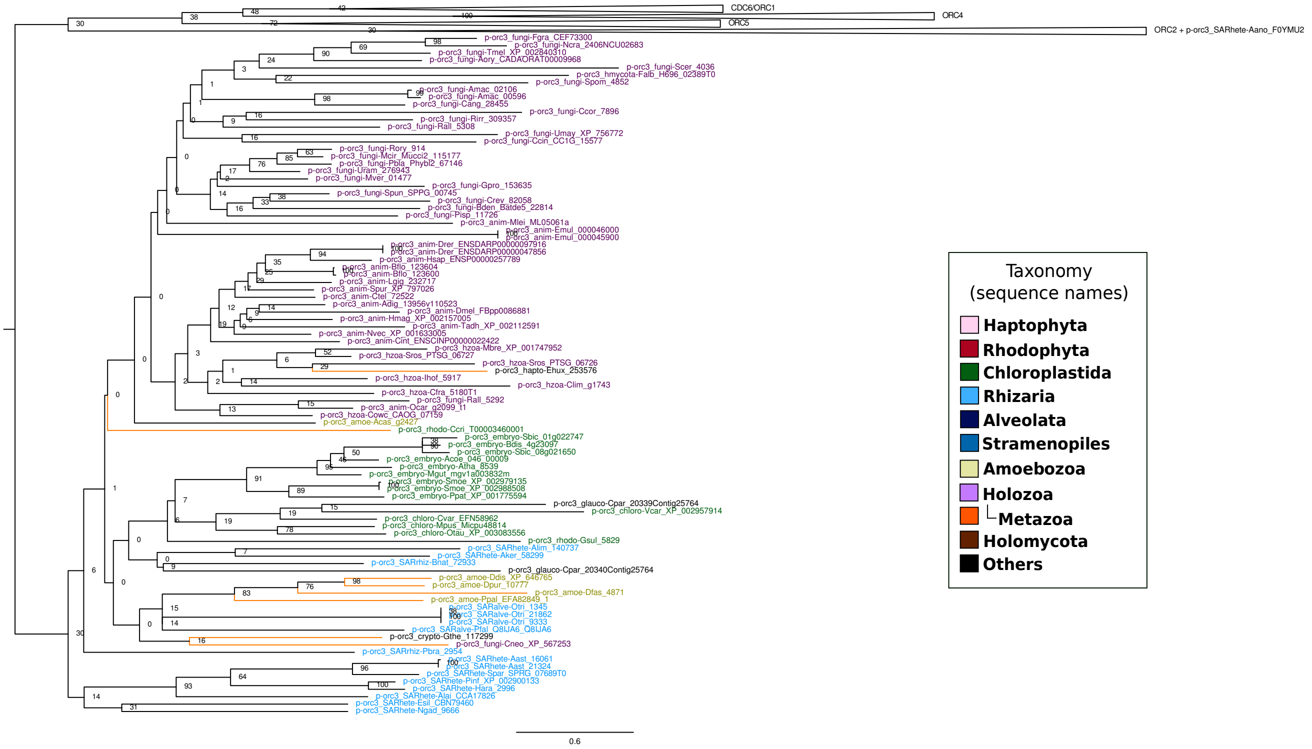

Supplementary Figure 7: Maximum-likelihood phylogenetic tree including all potential ORC4 orthologs identified in euk_db as well as the *bona fide* CDC6/ORC1 and ORC2-ORC3 and ORC5 sub_euk_db sequences identified, used for outgroups purposes. Nodal supports correspond to 100 rapid bootstrap replicates (RAxML). Sequence names start with a prefix indicating the pre-assigned ORC orthogroup using Blast and HMMER-based search approaches (e.g., p-orc4 corresponds to 'putative ORC4') and include the corresponding four-letter code species abbreviature (e.g., Hsap corresponds to *Homo sapiens*. See Supplementary Table 1 for the correspondence between the four-letter code and the species names). Sequence names were coloured according to the taxonomic colour code. ORC4 sequences were classified as *bona fide* members (black branches), putative members (orange branches), and unlikely members (red branches). For that, we took into account not only the phylogenetic position in the tree but also additional information such the Pfam protein domain architecture or the results from Blast alignments against other euk_db proteins.

Supplementary Figure 7

Phylogenetic classification of potential ORC4 proteins

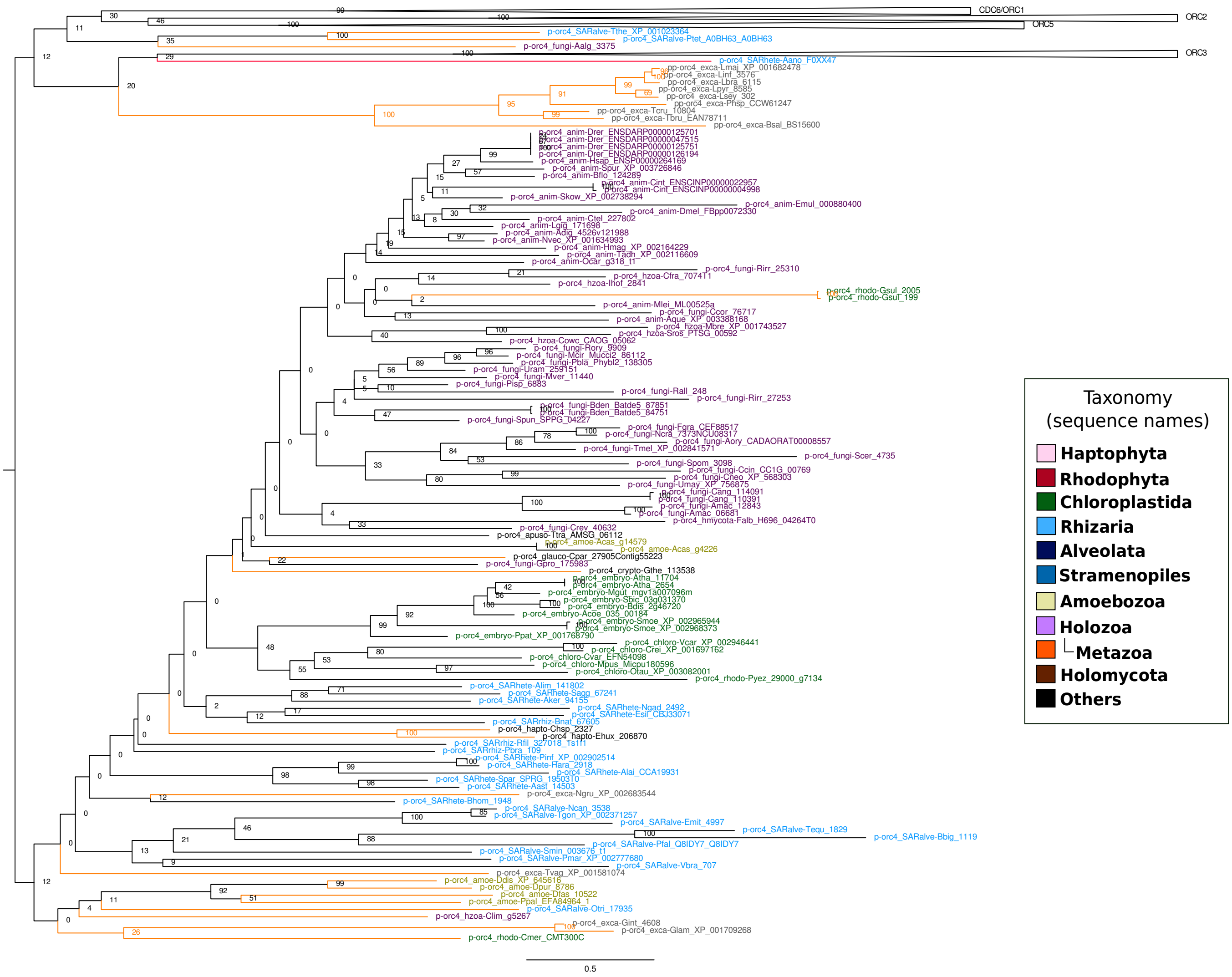

Supplementary Figure 8: Maximum-likelihood phylogenetic tree including all potential ORC5 orthologs identified in euk_db as well as the *bona fide* CDC6/ORC1 and ORC2-ORC4 sub_euk_db sequences identified, used for outgroups purposes. Nodal supports correspond to 100 rapid bootstrap replicates (RAxML). Sequence names start with a prefix indicating the pre-assigned ORC orthogroup using Blast and HMMER-based search approaches (e.g., p-orc5 corresponds to 'putative ORC5') and include the corresponding four-letter code species abbreviature (e.g. H,sap corresponds to *Homo sapiens*. See Supplementary Table 1 for the correspondence between the four-letter code and the species names). Sequence names were coloured according to the taxonomic colour code. ORC5 sequences were classified as *bona fide* members (black branches), putative members (orange branches), and unlikely members (red branches). For that, we took into account not only the phylogenetic position in the tree but also additional information such the Pfam protein domain architecture or the results from Blast alignments against other euk_db proteins.

Supplementary Figure 8

Phylogenetic classification of potential ORC5 proteins

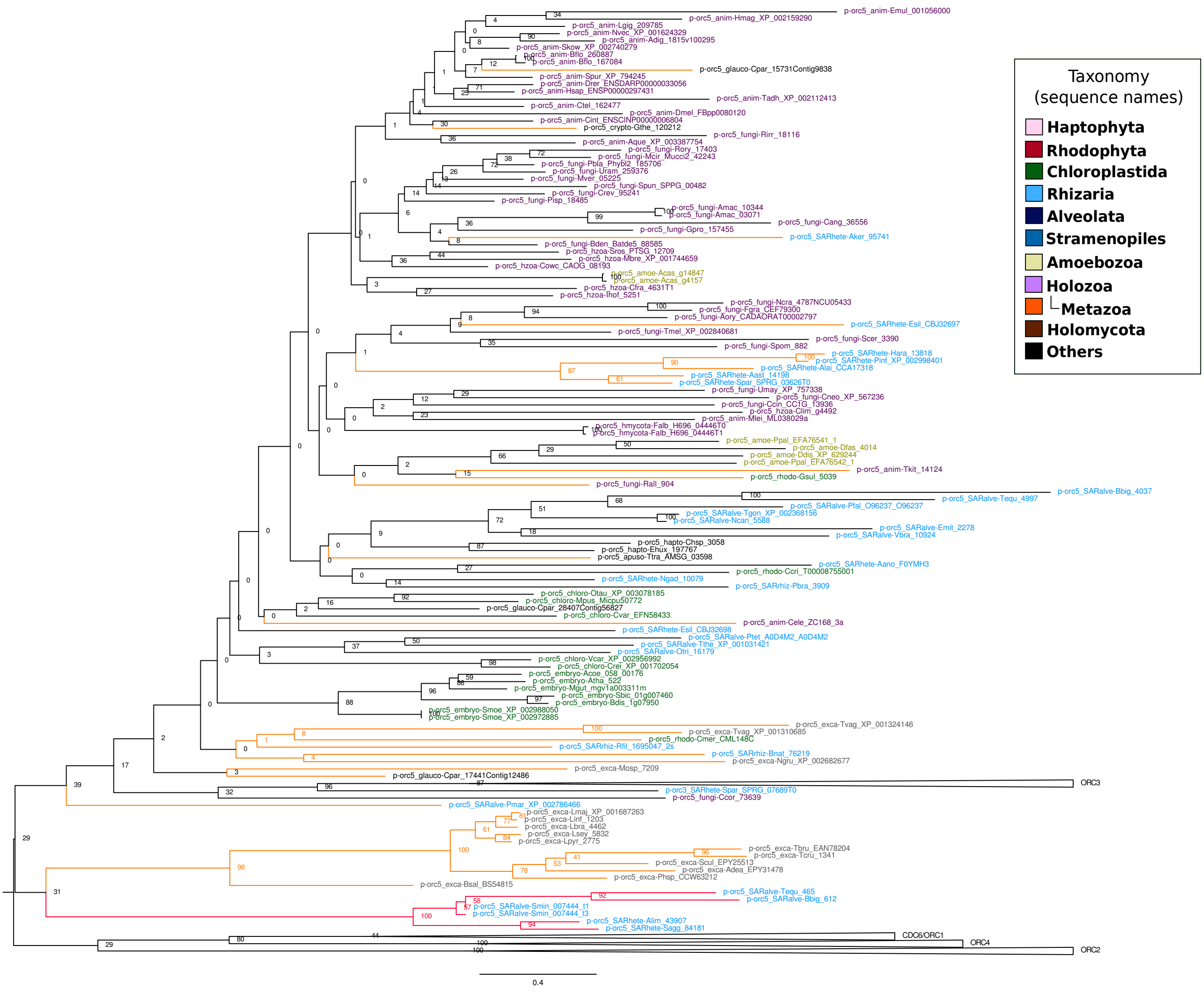

Supplementary Figure 9: Maximum-likelihood phylogenetic tree including all *bona fide* and likely CDC6/ORC1 orthologs identified in euk_db (see Supplementary Figure 1) as well as the sequences from the Archaeal ORC clade in Figure 1, used for outgroups purposes. Nodal supports correspond to 100 rapid bootstrap replicates (RAxML). Sequence names start with a prefix indicating the pre-assigned ORC orthogroup using Blast and HMMER-based search approaches (e.g., p-cdc6orc1 corresponds to 'putative CDC6/ORC1') and include the corresponding four-letter code species abbreviature (e.g., Hsap corresponds to *Homo sapiens*. See Supplementary Table 1 for the correspondence between the four-letter code and the species names). Sequence names were coloured according to the taxonomic colour code. Sequences were classified as *bona fide* ORC1 (light blue branches), *bona fide* CDC6 (purple branches). Sequence branching outside this two *bona fide* clades were considered as CDC6/ORC1 (dark blue branches). For that, we took into account not only the phylogenetic position in the tree but also additional information such the Pfam protein domain architecture or the results from Blast alignments against other euk_db proteins.

Supplementary Figure 9

Phylogenetic classification of potential CDC6 and ORC1 proteins

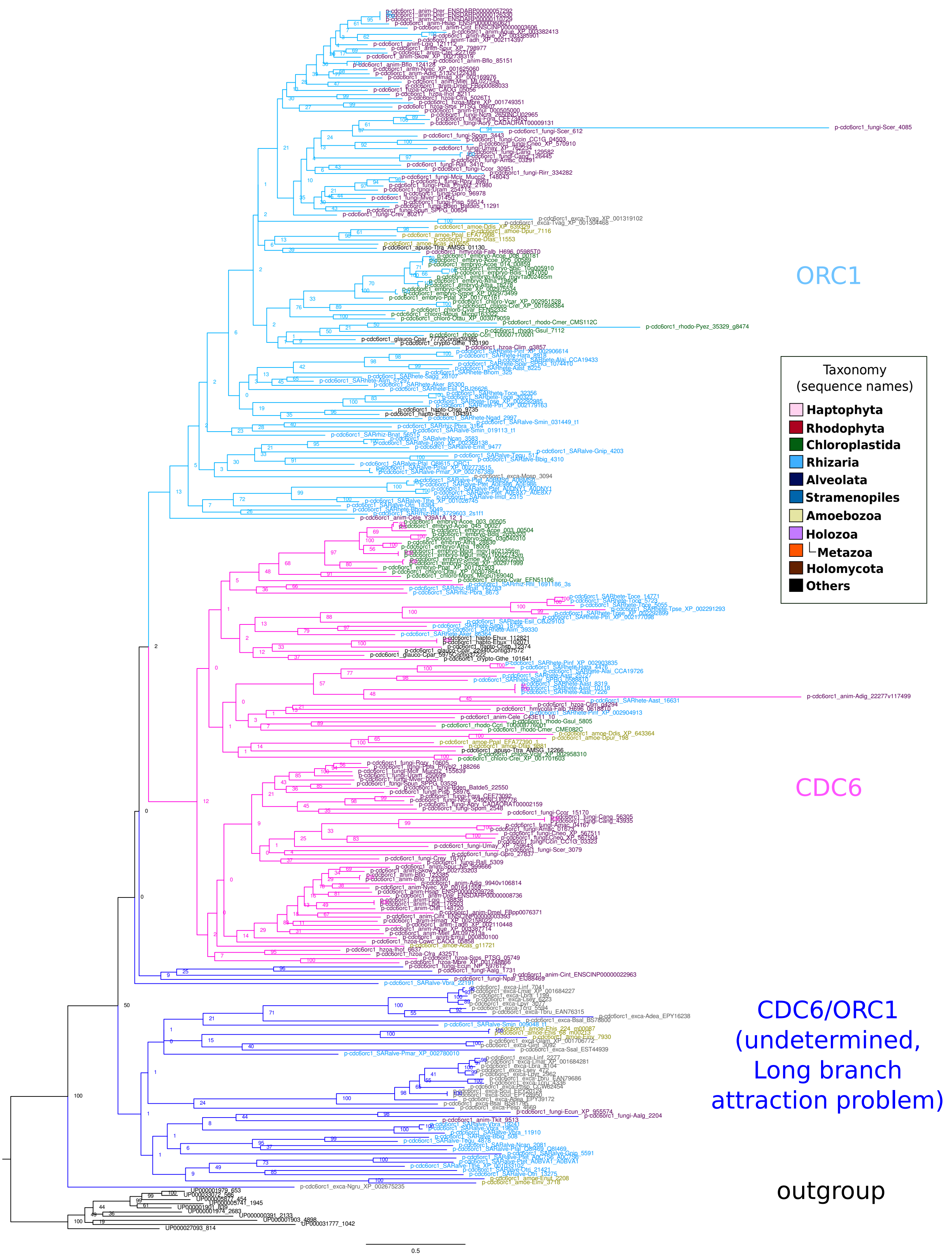

Supplementary Figure 10: (**A**) For every ORC subunit, the fraction of euk_db Amorphea, Diaphoretickes, Discoba, and Metamonada taxa from which we identified at least one *bona fide* ortholog (dark blue), not a *bona fide* but at least one likely ortholog (orange) or neither *bona fide* nor likely ortholog (red). See Figure 2 and Supplementary Table 1 for taxonomic information of euk_db taxa. (**B**) Potential scenarios for the diversification of CDC6+ORC1-5 from the ancestral CDC6/ORC1 archaeal-like gene according to four distinct possible eukaryotic phylogenies. Each circle represent a distinct CDC6+ORC1-5 subunit, and are coloured as shown in (**A**). Circles are drawn in branches where the origin of the specific subunit is proposed. Two distinct scenarios are evaluated for H2, H3 and H4 phylogenies (see main text).

Supplementary Figure 10

CDC6+ORC1-5 distribution
+ evolutionary scenarios proposed

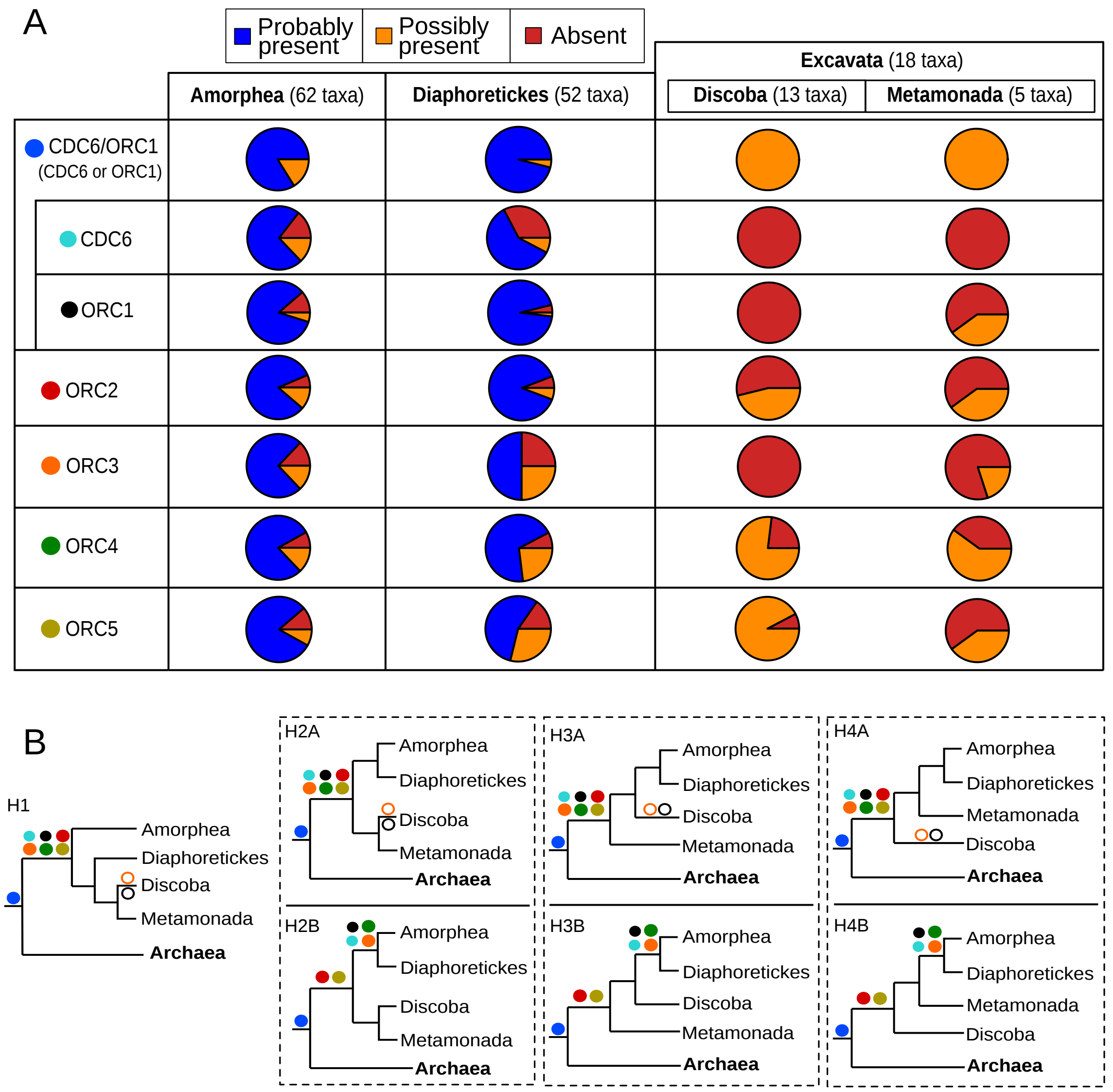

Supplementary Figure 11: Correlation analyses between genome size and number of ORIs metrics of 7 eukaryotes from which information is available. Metrics were plotted as raw counts and also in log10 scale. Pearson and Spearman correlation coefficients are also represented.

Supplementary Figure 11

Correlation analyses between
nº of ORIs and genome size

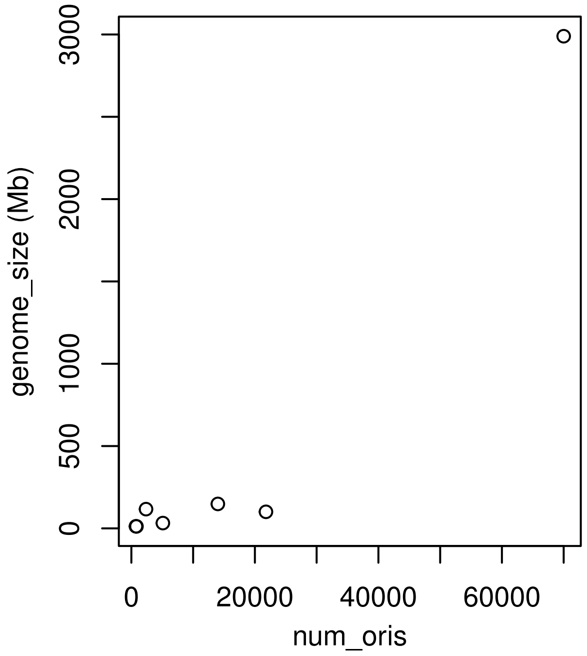

Homo sapiens 2989.43 Mb; ~70,000 ORIs
Arabidopsis thaliana 116.85 Mb; 2,374 ORIs
Drosophila melanogaster 148.50 Mb; 14,005 ORIs
Caenorhabditis elegans 100.73 Mb; 21,795 ORIs
Leishmania major 32.32 Mb; 5,100 ORIs
Schizosaccharomyces pombe 12.59 Mb; 741 ORIs
Saccharomyces cerevisiae 12.14 Mb; 829 ORIs

pearson = 0.96; spearman = 0.79

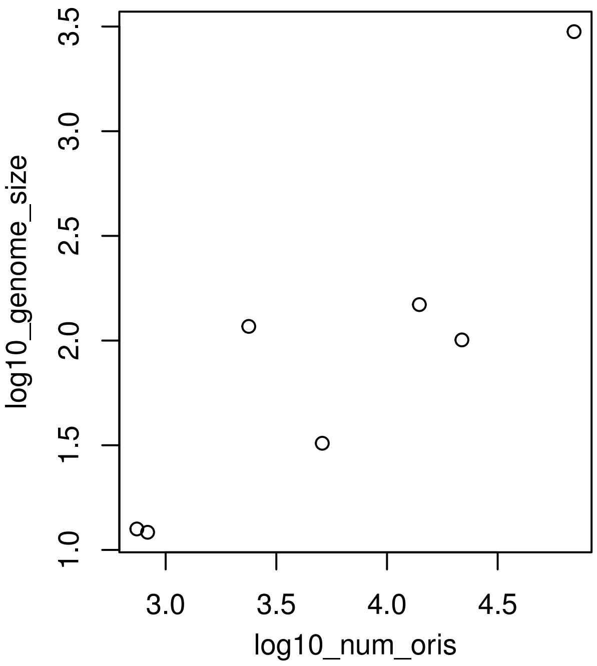

pearson = 0.88; spearman = 0.79

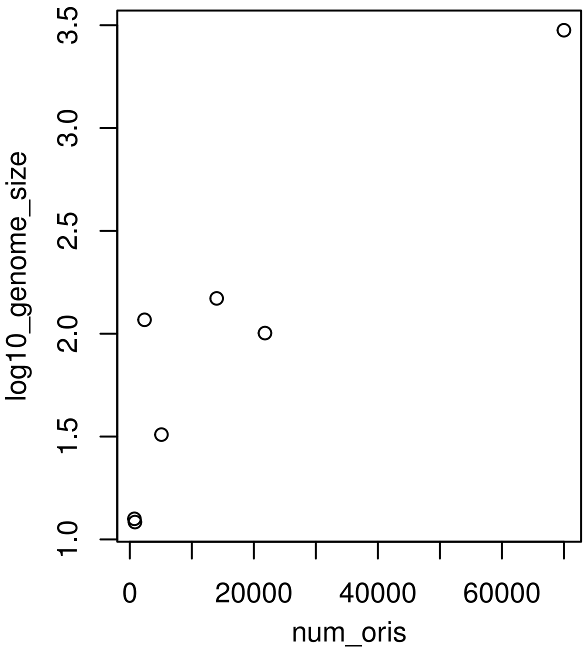

pearson = 0.91; spearman = 0.79

Supplementary Figure 12. Bayesian tree (PhyloBayes, 'PROTGAMMALG' model) including the CDC6 and ORC1-5 subunits from a subsampling of eukaryotic sequences (sub_euk_db) as well as archaeal sequences selected for rooting purposes. For the phylogeny, two chains were run until the maximum between chain discrepancy in bipartition frequencies fell below 0.3. Nodal supports correspond to Bayesian posterior probabilities (burn-in 25%)

Supplementary Figure 12 CDC6+ORC1-5 phylogeny (PhyloBayes)

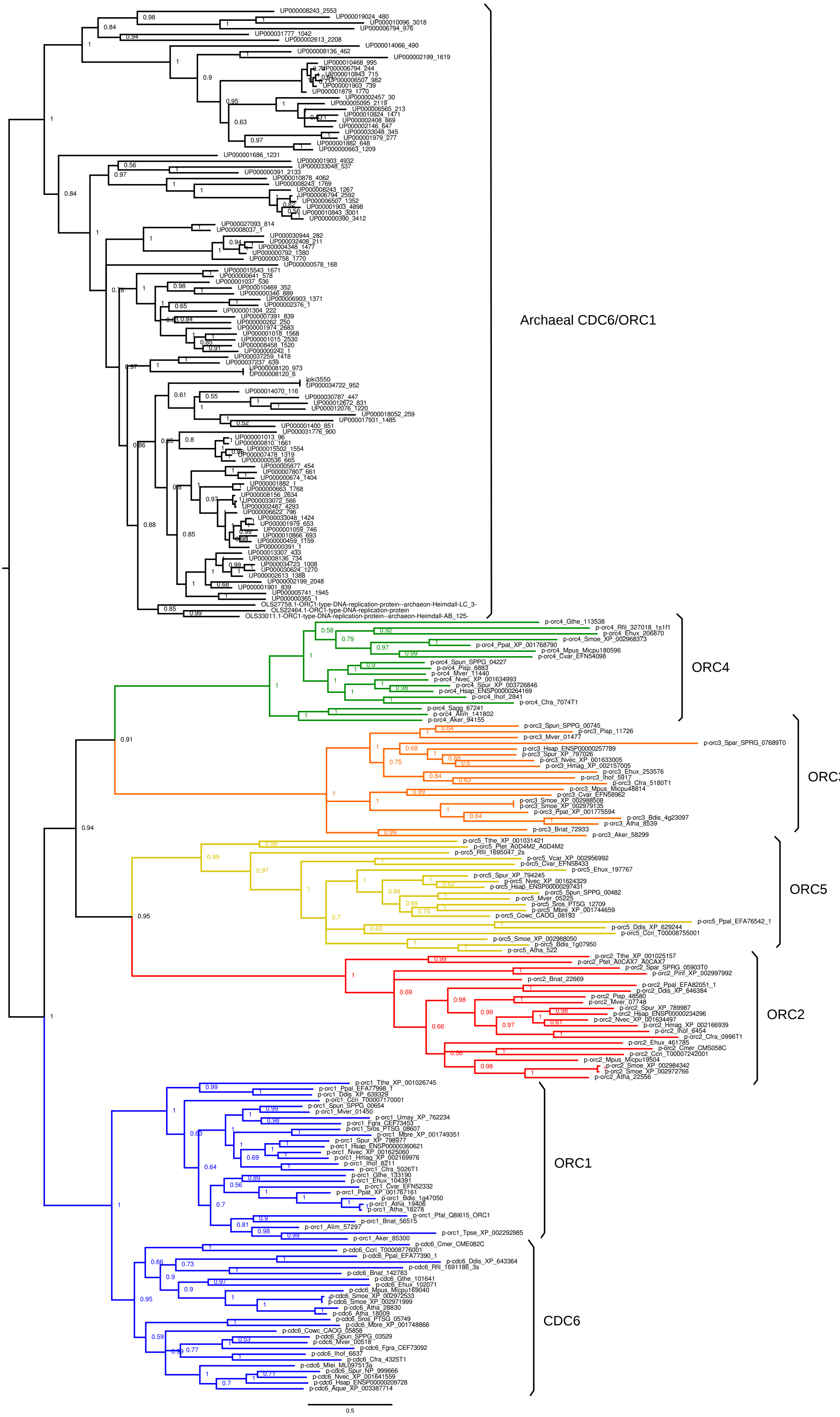

Supplement: evaa011_Supplementary_Data [file evaa011_supplementary_data.zip › supplementary_figures_151119.pdf]
