# Supplementary material for: Origin Recognition Complex (ORC) Evolution Is Influenced by Global Gene Duplication/Loss Patterns in Eukaryotic Genomes
Source: Genome Biol Evol. 2020 Jan 28;12(2):3878–89. doi: 10.1093/gbe/evaa011 (PMC7058166; doi:10.1093/gbe/evaa011)
Supplement: evaa011_Supplementary_Data [file evaa011_supplementary_data.zip › Ocaña-Pallarès ORC evol_MBE_SupplInfo.doc]

**Supplementary Data**

**Origin recognition complex (ORC) evolution is influenced by global gene duplication / loss patterns in eukaryotic genomes**

Eduard Ocaña-Pallarès^1^ ([0000-0001-6040-4216](https://orcid.org/0000-0001-6040-4216)), Zaida Vergara^2^ ([0000-0002-8810-1281](https://orcid.org/0000-0002-8810-1281)), Bénédicte Desvoyes^2^ (0000-0001-7116-9821), Manuel Tejada-Jimenez^3^ ([0000-0003-1702-6015](https://orcid.org/0000-0003-1702-6015)), Ainoa Romero-Jurado^3^ ([0000-0002-0048-2674](https://orcid.org/0000-0002-0048-2674)), Aurora Galvan^3^ ([0000-0002-7564-2281](https://orcid.org/0000-0002-7564-2281)), Emilio Fernandez^3^ ([0000-0001-5957-5392](https://orcid.org/0000-0001-5957-5392)), Iñaki Ruiz-Trillo^1,4,5^ ([0000-0001-6547-5304](http://orcid.org/0000-0001-6547-5304)), Crisanto Gutierrez^2^ ([0000-0001-8905-8222](http://orcid.org/0000-0001-8905-8222))

^1^ Institut de Biologia Evolutiva (CSIC-Universitat Pompeu Fabra), Passeig Marítim de la Barceloneta 37-49, 08003 Barcelona, Spain

^2^ Centro de Biologia Molecular Severo Ochoa, CSIC-UAM, Nicolas Cabrera 1, Cantoblanco, 28049 Madrid, Spain

^3^ Departamento de Bioquímica y Biología Molecular, Facultad de Ciencias, Universidad de Córdoba, Campus de Rabanales, 14071 Córdoba, Spain

^4^ Departament de Genètica, Microbiologia i Estadística, Universitat de Barcelona, Av. Diagonal, 645, 08028 Barcelona, Spain

^5^ ICREA, Passeig Lluís Companys 23, 08010, Barcelona, Spain

+ Correspondence to [inaki.ruiz@ibe.upf-csic.es](mailto:inaki.ruiz@ibe.upf-csic.es) or [cgutierrez@cbm.csic.es](mailto:cgutierrez@cbm.csic.es)

Keywords: Origin recognition complex (ORC), DNA replication, eukaryotic evolution, centriole, gene loss, parasitism

Running title: Evolution of origin recognition complex (ORC) in eukaryotes

**Supplementary Information Content**

- Supplementary figures 1 – 12
- Supplementary tables 1 – 2 (separate files)
- Supplementary Results
- Supplementary Methods
