# Supplementary material for: Origin Recognition Complex (ORC) Evolution Is Influenced by Global Gene Duplication/Loss Patterns in Eukaryotic Genomes
Source: Genome Biol Evol. 2020 Jan 28;12(2):3878–89. doi: 10.1093/gbe/evaa011 (PMC7058166; doi:10.1093/gbe/evaa011)
Supplement: evaa011_Supplementary_Data [file evaa011_supplementary_data.zip › Ocaña-Pallarès ORC evol_SupplMethods_151119.doc]

**Supplementary Methods**

**Origin recognition complex (ORC) evolution is influenced by global gene duplication / loss patterns in eukaryotic genomes**

Eduard Ocaña-Pallarès^1^ ([0000-0001-6040-4216](https://orcid.org/0000-0001-6040-4216)), Zaida Vergara^2^ ([0000-0002-8810-1281](https://orcid.org/0000-0002-8810-1281)), Bénédicte Desvoyes^2^ (0000-0001-7116-9821), Manuel Tejada-Jimenez^3^ ([0000-0003-1702-6015](https://orcid.org/0000-0003-1702-6015)), Ainoa Romero-Jurado^3^ ([0000-0002-0048-2674](https://orcid.org/0000-0002-0048-2674)), Aurora Galvan^3^ ([0000-0002-7564-2281](https://orcid.org/0000-0002-7564-2281)), Emilio Fernandez^3^ ([0000-0001-5957-5392](https://orcid.org/0000-0001-5957-5392)), Iñaki Ruiz-Trillo^1,4,5^ ([0000-0001-6547-5304](http://orcid.org/0000-0001-6547-5304)), Crisanto Gutierrez^2^ ([0000-0001-8905-8222](http://orcid.org/0000-0001-8905-8222))

^1^ Institut de Biologia Evolutiva (CSIC-Universitat Pompeu Fabra), Passeig Marítim de la Barceloneta 37-49, 08003 Barcelona, Spain

^2^ Centro de Biologia Molecular Severo Ochoa, CSIC-UAM, Nicolas Cabrera 1, Cantoblanco, 28049 Madrid, Spain

^3^ Departamento de Bioquímica y Biología Molecular, Facultad de Ciencias, Universidad de Córdoba, Campus de Rabanales, 14071 Córdoba, Spain

^4^ Departament de Genètica, Microbiologia i Estadística, Universitat de Barcelona, Av. Diagonal, 645, 08028 Barcelona, Spain

^5^ ICREA, Passeig Lluís Companys 23, 08010, Barcelona, Spain

+ Correspondence to [inaki.ruiz@ibe.upf-csic.es](mailto:inaki.ruiz@ibe.upf-csic.es) or [cgutierrez@cbm.csic.es](mailto:cgutierrez@cbm.csic.es)

Keywords: Origin recognition complex (ORC), DNA replication, eukaryotic evolution, centriole, gene loss, parasitism

Running title: Evolution of origin recognition complex (ORC) in eukaryotes

**Screening of ORC family member orthologs**

We performed a first search of potential CDC6, ORC1, ORC2, ORC3, ORC4 and ORC5 members (CDC6+ORC1-5, hereafter referred as ORC subunits) using *Blast* (Altschul, et al. 1990) and *Hidden Markov Model* (HMM) (Eddy 2011) approaches. For the *Blast* approach, we first downloaded (from NCBI) the ORC sequences from three reference eukaryotes (reference ORC sequences): *Homo sapiens*, *Saccharomyces cerevisiae,* and *Arabidopsis thaliana*. We then separated the sequences of the three species in different FASTA files and used them to query a database consisting of 132 publicly available eukaryotic proteomes generated from genome projects (euk_db, see Supplementary Table 1) using *BLASTP* v2.3.0+ [-evalue 1e-3; only the non-default software commands will be specified]. All the aligned sequences were retrieved from euk_db and aligned back to the proteomes of these three species to keep as potential orthologs those sequences whose best hit was against an ORC. We also complemented this first screening of potential orthologs by scanning the euk_db sequences for the characteristic protein domain architectures of each ORC subunits (HMM approach). For that, we first obtained all the protein domain architectures for all euk_db sequences with *PfamScan* (Finn, et al. 2014) using Pfam A v29. Then, based on the Pfam domains annotated for the reference ORC sequences, we considered as potential orthologs all euk_db proteins with the following Pfam domains: *Cdc6_C* for CDC6; *AAA* and *PHD* or *BAH* for ORC1; *ORC2* for ORC2; *ORC3_N* for ORC3; *ORC4_C* for ORC4 and *ORC5_C* for ORC5.

We then performed a second and more sophisticated approach to capture potential orthologs that may had remained undetected. We grouped the potential orthologs obtained from *Blast* and *PfamScan* searches in six FASTA files, one per ORC subunit. Then, each FASTA file was aligned to all euk_db proteomes in which no potential members for that subunits were detected [*BLASTP*: -evalue 1e-3]. The best scoring target of every proteome were subsequently aligned to subunit-specific databases, each one including all protein sequences (i.e. ORC and non-ORC) of all the proteomes in which at least one potential subunits member was found in the initial searches. We incorporated into the set of potential orthologs those proteins (i) whose best hit in the subunit-specific database was a potential ORC sequence, (ii) that were not previously classified as potential ORC, and (iii) whose Pfam domain architecture is not clearly distinct to that of the reference ORC sequences (see above). Lastly, because it was reported the existence of a divergent ORC4 ortholog sequence in *Trypanosoma brucei* (Tiengwe, et al. 2012), we used the *Tbru_EAN78711* sequence to query euk_db for the potential presence of further remote ORC4 orthologs in other Excavata [*BLASTP*: -evalue 1e-3]. The eight sequences aligned, all from other Excavata species, were incorporated into the potential ORC4 subunit file.

**Constructing the phylogenetic trees**

Phylogenetic relationships between ORC subunits

We reconstructed the evolutionary history of the ORC protein family using only a subset of the proteomes represented in euk_db (sub_euk_db). Species names in bold in Supplementary Table 1 are the ones included in sub_euk_db. The main criteria to decide which proteomes (sub_euk_db) were considered for the iterative phylogenetic inference process was the aim of covering the existent genomic diversity of the eukaryotic tree. Therefore, for each eukaryotic group, we chose as representatives those proteomes in which we identified potential orthologs for all or most of the six ORC subunits. However, Excavata taxa was not included because we observed in exploratory trees that their sequences were highly divergent compared to the other eukaryotes. Sequences from SAR taxa (Stramenopiles + Alveolata + Rhizaria), also highly divergent, were incorporated but not in the initial iterations (see below).

We retrieved the potential orthologs identified for the sub_euk_db taxa and constructed separate phylogenetic trees for each ORC subunit. From this initial set of trees, we performed a sequential iterative process consisting in (i) pruning highly divergent sequences, (ii) inspecting the resulting trees, (iii) adding SAR taxa, (iv) pruning highly divergent SAR taxa and (v) a final inspection of the resulting tree. Once we obtained representative and curated datasets for each ORC subunit, we combined them and performed a second iterative process now with all subunits together. In this second iterative process, we pruned sequences that did not unambiguously group with the subunit that was assigned (see *Screening of ORC family member orthologs* section). Most of the phylogenetic trees of this study were constructed with the following procedure: first, protein sequences were aligned with *MAFFT* v7.123b (Katoh, et al. 2002) [mafft-einsi] and alignments were trimmed with *trimAl* v1.4.rev15 (Capella-Gutierrez, et al. 2009) using the -gappyout option. Maximum likelihood phylogenetic inferences were done using *RAxML* v8.2.4 (Stamatakis 2014) with rapid bootstrap analysis (100 replicates). The model for the phylogenetic inference was selected according to BIC criteria with *ProtTest* v3.2 (Darriba, et al. 2011). In the following sections, only the methods of those phylogenetic inference processes that differ from this general procedure will be specified. The alignments used to construct the phylogenies represented in Figures and Supplementary Figures are available at <https://figshare.com/s/d09531ca7fabd3b88f27>. All sequences included in the alignments are also available (*orc.draft.fa*).

We used the curated ORC dataset obtained in the step above to reconstruct the evolutionary history of the ORC protein family. In this phylogenetic inference process, we incorporated prokaryotic sequences to be used as outgroup. We captured those prokaryotic sequences from a database (prok_db) constructed from Uniprot bacterial and archaeal reference proteomes (Release 2016_02) (Apweiler, et al. 2004). To query prok_db, we used all the potential ORC subunit members of sub_euk_db [*BLASTP*: -evalue 1e-3, -max_target_seqs 10], and only the protein regions corresponding to the ORC-related Pfam domains (see *Identifying ORC family member orthologs* section). The 10 best hits of each query in prok_db were put together with the potential orthologs from sub_euk_db. In the phylogenetic reconstruction, instead of using the rapid bootstrap method, we computed in separate *RAxML* runs the best tree [-# 100] and the bootstrap trees [-N 100] using in both cases the gamma model of rate heterogeneity and LG amino acid substitution matrix [from *ProtTest* results]. Then, the two output files were merged to construct the final bipartitions tree. A Bayesian phylogeny was also reconstructed with PhyloBayes-MPI v1.8 under the ‘LG+gamma4’ model (Lartillot, et al. 2013). For this, two chains were ran until the maximum between chain discrepancy in bipartition frequencies fell below 0.3 (burn-in 25%).

Phylogenetic-based subunit classification of *bona fide* subunit orthologs

For each ORC subunit, we constructed a phylogenetic tree including all the putative sequences identified to belong to that group (Supplementary Figures 4-8). The trees were manually inspected and sequences were classified as ‘*bona fide*’ subunit members (black branches), ‘likely’ subunit members (orange branches), and ‘unlikely’ subunit members (red branches). For that, phylogenetic information from the tree was considered but also additional information such as the protein domain architecture or the results from *Blast* alignments to other euk_db proteins. Given the similarity between CDC6 and ORC1 subunit, the most divergent proteins of these two families could not be unambiguously classified into one of the two families (see orange branches Supplementary Figure 8). Therefore, for these two subunits (CDC6 and ORC1), we first identified those *bona fide* CDC6/ORC1 proteins in a first phylogenetic tree, and we then performed a second tree to discern between CDC6 and ORC1 proteins (Supplementary Figure 9). This second tree included as outgroup the sequences from the Archaeal ORC clade in Figure 1 instead of the other eukaryotic ORC subunits (the last was done for ORC2-5 trees). In this case, we decided to include only one outgroup in order to reduce as much as possible the divergence introduced by the outgroup sequences. We chose the Archaeal ORC as an outgroup because the sequences from this clade have, in average, the lowest branch length distance to CDC6/ORC1 sequences in Figure 1A tree (distances between terminal nodes were retrieved using the *cophenetic.phylo* function from the R *ape* package (Paradis, et al. 2004)).

Identification of false paralogues

We found that some proteomes in euk_db have multiple isoforms per gene, which could artifactually inflate the number of paralogs for each ORC subunit. For every species, we considered only the sequences classified as ‘*bona fide*’ and ‘likely’ subunit members, and we generated a FASTA file with those putative paralogs. Each FASTA file was aligned to the corresponding species genome [*TBLASTN*: -evalue 1e-3] and those putative species-specific paralogs whose best scoring hit corresponded to overlapping genomic regions considered to be isoforms of a same gene (i.e. false paralogs). Among the different isoforms, we only kept the one aligning with the highest score to the reference ORC sequences [*BLASTP*: -evalue 1e-3]. The final ORC subunit counts per species is available in Supplementary Table 1. All the ORC sequences identified (excluding false paralogs) are available at <https://figshare.com/s/d09531ca7fabd3b88f27> (*orc.clean.fa*).

Detection of unannotated and highly divergent ORC proteins

We also used *TBLASTN* to search for potential unannotated ORC sequences in the genomes of those taxa with missing subunits in euk_db [*TBLASTN*: -evalue 1e-3], using as queries the reference ORC sequences. We also used custom HMM of every ORC subunit to search for potential highly divergent sequences that may had not been detected by *Blast* searches. The custom HMM of every subunit was constructed using the corresponding ‘*bona fide*’ and ‘likely’ members identified from euk_db [*hmmbuild*] (available at <https://figshare.com/s/d09531ca7fabd3b88f27>). We then scanned euk_db using a database including all custom HMMs (customHMM_db) [*hmmscan* -E 0.1]. For every subunit, hits from those species in which we already identified a ‘*bona fide*’ or ‘likely’ member/s were classified as True positives (‘TP’) or False positive (‘FP’), depending on whether the protein was identified or not as a ‘*bona fide*’ or ‘likely’ member. Hits from species without a subunit member identified were classified as ‘Uncertain’. For every subunit, we computed the score cutoff value in which only 5% of ‘FP’ would be detected as ‘TP’. All ‘Uncertain’ hits with a score higher than the cutoff were considered as candidate subunit members. The identity of 17 and 18 candidate subunit members identified through *TBLASTN* and HMM searches, respectively, was validated through phylogenetic analyses. Trees included all ‘*bona fide*’ and ‘likely’ members of the corresponding subunit. As outgroups, representatives of other ORC subunits were included, and also the non-ORC sequences from *H. sapiens*, *A. thaliana*, and *S. cerevisiae* that showed a positive hit against the corresponding custom HMM were included.

**Analyses of ORC distribution in relation to parasitic lifestyle and genomic features**

Clustering of sampled eukaryotes from euk_db according to the ORC subunit counts was done with the *heatmap.2* function from the R *gplots* package. The same package was used to plot heatmaps of ORC subunit counts, genome size and gene content metrics. Given the high differences in genome size between eukaryotes, raw length measures (in Mb) were transformed in log10 scale and normalized to the largest genome length found in euk_db. Gene content metrics were obtained from the number of sequences found per taxa in euk_db, and were also normalized to the largest value found. Spearman correlation metrics were computed with *cor.test* R function, boxplots with *boxplot* R function and Mann-Whitney U test with *wilcox.test* R function. We used Mann-Whitney U test instead of Student's t-test because the distributions were not normal according to histogram plots.

**Phylogenetic analyses of PHD domain in Chloroplastida ORC1 sequences**

The protein regions corresponding to the *PHD* Pfam domains from ORC1 sequences of Chloroplastida (queries) were aligned to euk_db and to a comprehensive prokaryotic database (prok_db, see Supplementary Material) [*BLASTP*: -evalue 1e-3], and the 10 best scoring hits of every query were kept (targets). No hits were found in prok_db searches. Query and target sequences were aligned and a phylogenetic tree was constructed and represented using *ITOL* (Letunic and Bork 2016). Domain architectures identified through *PfamScan* are also represented.

***Chlamydomonas reinhardtii* culture conditions**

*C. reinhardtii* cells were cultured in Tris‐acetate‐phosphate (TAP) containing 8 mM ammonium chloride or 25 mM potassium nitrate, at 25 °C under continuous light and agitation, till exponential growth phase. Algal cell number was evaluated by a cell counter system (Sysmex F500, Sysmed Lab Inc.).

**Generation of amiRNA ORC1 strains and transformation**

The promoter of the *C. reinhardtii NITRATE REDUCTASE* (*NR*) was cloned from the pNR-Nar3TNR-1 vector using KOD hot start DNA polymerase (71086 – Merck Millipore) and the primers listed in Supplementary Table 2. The fragment was inserted into the vector pChlamyRNA3 (Molnar, et al. 2009) using the restriction enzymes AvrII and NdeI (NEB and Thermo Scientific), to obtain a scaffold plasmid pChlamyNR-RNA3 containing the *C. reinhardtii* MIR1157 under the control of the *NR* promoter. The transcript sequence of *CrORC1* (Cre10.g455600.t1.1) was retrieved from Phytozome11 ([https://phytozome.jgi.doe.gov/pz/portal.html#!info?alias=Org_Creinhardtii](https://phytozome.jgi.doe.gov/pz/portal.html#!info?alias=Org_Creinhardtii)) and used in the Web MicroRNA Designer platform (WMD3; <http://wmd3.weigelworld.org/cgi-bin/webapp.cgi>) to design a specific amiRNA. The custom sequence was introduced in the pChlamyNR-RNA3 vector according to (Molnar, et al. 2009). Briefly, 16.7 µM of each ssDNA primer was boiled for 5 min and annealed at room temperature overnight in 13.3 mM Tris-HCl pH 8.0, 1.3 mM EDTA pH 8.0, 66.7 mM NaCl buffer. The dsDNA amiRNA was introduced into the pChlamyNR-RNA3 vector by digestion with SpeI (Thermo Scientific) restriction enzyme. The *C. reinhardtii* strain 704 (Loppes, et al. 1999) was transformed using the glass beads method (Kindle 1990) with some modifications: concentration of polyethylene glycol 8000 was 2.5 % (w/v); cells were shaken for 30 s, DNA concentration was adjusted to 1 μg/transformation reaction. Transformants were selected in TAP medium containing ammonium as nitrogen source and supplemented with 25 μg/mL paromomycin.

**Quantitative Real-Time PCR**

Quantification of ORC1 transcripts was carried out by quantitative-PCR using primers indicated in Supplementary Table 2. Reverse transcription of 0.5 µg of total RNA was performed using iScript cDNA synthesis kit (Bio-Rad) following manufacturer instructions. qPCR was carried out on an iCycler iQreal-time PCR detection system (Bio-Rad) using Ssfast Mix (Bio-Rad) as fluorescent dye. RNA levels were normalized by using the ubiquitin-ligase gene as internal standard (Gonzalez-Ballester, et al. 2004).

**Motility assay**

*C. reinhardtii* cells were cultured in TAP medium containing ammonium as nitrogen source till exponential growth phase, washed three times with nitrogen-free medium, and then transferred to the corresponding medium (8 mM ammonium chloride or 25 mM potassium nitrate) for 24h. Subsequently, motility was analysed under optical microscope (Leica DM750) as the number of cells crossing a square of a Neubauer chamber during 10 s, compared to not-moving cells within the same square.

**Immunocytochemistry of *C. reinhardtii* cells**

Algal cells were cultured in liquid TAP media containing 8 mM ammonium chloride till exponential growth phase. Then cells were washed three times with nitrogen-free TAP medium and subsequently transferred to the appropriate incubation conditions. After incubation cells were harvested by centrifugation at 4000 x *g* for 2 min. Then cells were resuspended in microtubule-stabilizing buffer (MTSB, 50 mM PIPES-KOH pH 6.9, 5 mM EGTA, 5 mM MgSO4), adjusting cell density to 10^7^ cells/ml. 100 µl of cells were placed on a poly-L-lysine-coated slide and incubated for 10 min at room temperature. Cells were fixed by adding 4 % paraformaldehyde in MTSB and incubation for 10 min at room temperature. Slides were washed three times with MTSB. In order to eliminate chlorophyll autofluorescence slides were submerged twice into a Coplin jar filled with methanol at -20 ºC for 10 min. Finally, slides were air-dried and stored at -20 ºC. Immunofluorescence was performed as described (Uniacke, et al. 2011), with minor modifications. Cells were rehydrated with PBS and permeabilized with PBS- 2% Triton X-100 for 10 min at room temperature (RT). After blocking with PBS - 3% BSA, 10% horse serum, slides were incubated overnight at 4ºC with anti-acetylated tubulin antibody (Clone 6-11B-1, Sigma, dilution 1:1000) and 1 h at RT with daylight 488 horse anti-mouse antibody (Vector Laboratories DI-2488, 1:500). Nuclei were counterstained with DAPI and slides mounted with Citifluor. Observations were carried out with a confocal microscope Zeiss LSM800 and images processed with FIJI-ImageJ.

**References**

Altschul SF, Gish W, Miller W, Myers EW, Lipman DJ 1990. Basic local alignment search tool. J Mol Biol 215: 403-410.

Apweiler R, et al. 2004. UniProt: the Universal Protein knowledgebase. Nucleic Acids Res 32: D115-119.

Capella-Gutierrez S, Silla-Martinez JM, Gabaldon T 2009. trimAl: a tool for automated alignment trimming in large-scale phylogenetic analyses. Bioinformatics 25: 1972-1973.

Darriba D, Taboada GL, Doallo R, Posada D 2011. ProtTest 3: fast selection of best-fit models of protein evolution. Bioinformatics 27: 1164-1165.

Eddy SR 2011. Accelerated Profile HMM Searches. PLoS Comput Biol 7: e1002195.

Finn RD, et al. 2014. Pfam: the protein families database. Nucleic Acids Res 42: D222-230.

Gonzalez-Ballester D, Camargo A, Fernandez E 2004. Ammonium transporter genes in Chlamydomonas: the nitrate-specific regulatory gene Nit2 is involved in Amt1;1 expression. Plant Mol Biol 56: 863-878.

Katoh K, Misawa K, Kuma K, Miyata T 2002. MAFFT: a novel method for rapid multiple sequence alignment based on fast Fourier transform. Nucleic Acids Res 30: 3059-3066.

Kindle KL 1990. High-frequency nuclear transformation of Chlamydomonas reinhardtii. Proc Natl Acad Sci U S A 87: 1228-1232.

Lartillot N, Rodrigue N, Stubbs D, Richer J 2013. PhyloBayes MPI: phylogenetic reconstruction with infinite mixtures of profiles in a parallel environment. Syst Biol 62: 611-615. doi: 10.1093/sysbio/syt022

Letunic I, Bork P 2016. Interactive tree of life (iTOL) v3: an online tool for the display and annotation of phylogenetic and other trees. Nucleic Acids Res 44: W242-245. doi: 10.1093/nar/gkw290

Loppes R, Radoux M, Ohresser MC, Matagne RF 1999. Transcriptional regulation of the Nia1 gene encoding nitrate reductase in Chlamydomonas reinhardtii: effects of various environmental factors on the expression of a reporter gene under the control of the Nia1 promoter. Plant Mol Biol 41: 701-711.

Molnar A, et al. 2009. Highly specific gene silencing by artificial microRNAs in the unicellular alga Chlamydomonas reinhardtii. Plant J 58: 165-174.

Paradis E, Claude J, Strimmer K 2004. APE: Analyses of Phylogenetics and Evolution in R language. Bioinformatics 20: 289-290.

Stamatakis A 2014. RAxML version 8: a tool for phylogenetic analysis and post-analysis of large phylogenies. Bioinformatics 30: 1312-1313.

Tiengwe C, et al. 2012. Identification of ORC1/CDC6-interacting factors in Trypanosoma brucei reveals critical features of origin recognition complex architecture. PLoS One 7: e32674.

Uniacke J, Colon-Ramos D, Zerges W 2011. FISH and immunofluorescence staining in Chlamydomonas. Methods Mol Biol 714: 15-29.
