# Supplementary material for: Origin Recognition Complex (ORC) Evolution Is Influenced by Global Gene Duplication/Loss Patterns in Eukaryotic Genomes
Source: Genome Biol Evol. 2020 Jan 28;12(2):3878–89. doi: 10.1093/gbe/evaa011 (PMC7058166; doi:10.1093/gbe/evaa011)
Supplement: evaa011_Supplementary_Data [file evaa011_supplementary_data.zip › Ocaña-Pallarès ORC evol_SupplResults.151119.doc]

**Supplementary Results**

**Origin recognition complex (ORC) evolution is influenced by global gene duplication / loss patterns in eukaryotic genomes**

Eduard Ocaña-Pallarès^1^ ([0000-0001-6040-4216](https://orcid.org/0000-0001-6040-4216)), Zaida Vergara^2^ ([0000-0002-8810-1281](https://orcid.org/0000-0002-8810-1281)), Bénédicte Desvoyes^2^ (0000-0001-7116-9821), Manuel Tejada-Jimenez^3^ ([0000-0003-1702-6015](https://orcid.org/0000-0003-1702-6015)), Ainoa Romero-Jurado^3^ ([0000-0002-0048-2674](https://orcid.org/0000-0002-0048-2674)), Aurora Galvan^3^ ([0000-0002-7564-2281](https://orcid.org/0000-0002-7564-2281)), Emilio Fernandez^3^ ([0000-0001-5957-5392](https://orcid.org/0000-0001-5957-5392)), Iñaki Ruiz-Trillo^1,4,5^ ([0000-0001-6547-5304](http://orcid.org/0000-0001-6547-5304)), Crisanto Gutierrez^2^ ([0000-0001-8905-8222](http://orcid.org/0000-0001-8905-8222))

^1^ Institut de Biologia Evolutiva (CSIC-Universitat Pompeu Fabra), Passeig Marítim de la Barceloneta 37-49, 08003 Barcelona, Spain

^2^ Centro de Biologia Molecular Severo Ochoa, CSIC-UAM, Nicolas Cabrera 1, Cantoblanco, 28049 Madrid, Spain

^3^ Departamento de Bioquímica y Biología Molecular, Facultad de Ciencias, Universidad de Córdoba, Campus de Rabanales, 14071 Córdoba, Spain

^4^ Departament de Genètica, Microbiologia i Estadística, Universitat de Barcelona, Av. Diagonal, 645, 08028 Barcelona, Spain

^5^ ICREA, Passeig Lluís Companys 23, 08010, Barcelona, Spain

+ Correspondence to [inaki.ruiz@ibe.upf-csic.es](mailto:inaki.ruiz@ibe.upf-csic.es) or [cgutierrez@cbm.csic.es](mailto:cgutierrez@cbm.csic.es)

Keywords: Origin recognition complex (ORC), DNA replication, eukaryotic evolution, centriole, gene loss, parasitism

Running title: Evolution of origin recognition complex (ORC) in eukaryotes

**Origin of the eukaryotic ORC**

Inferring the ancestral state of ORC in eukaryotes has always been controversial due to uncertainties related to the subunit configuration of ORC in Excavata (Tiengwe, et al. 2012; Marques, et al. 2016) (represented by Discoba and Metamonada in euk_db). Moreover, the phylogenetic position of Excavata with respect to Amorphea (Amoebozoa, Opisthokonta and others, see taxonomic groups in Fig. 2A) and Diaphoretickes (Archaeplastida, SAR and others), as well as the monophyly of Excavata, remain controversial (Adl, et al. 2019). Initial results proposed that trypanosomatids (Discoba) only have a single functional CDC6/ORC1 protein (Godoy, et al. 2009), possibly conserving the ancestral archaeal-like state (Cavalier-Smith 2010). However, later studies identified three other components of the complex: a divergent ORC4-like protein (Tiengwe, et al. 2012) and two highly divergent proteins that were proposed to be potential ORC2 and ORC5 orthologs given similarities proposed to be only detectable at structural level (Marques, et al. 2016). Our results confirm the identity of ORC5 and ORC2-like proteins in *Trypanosoma*. In particular, we identified the ORC5-like proteins in the initial *Blast* and *PfamScan* searches, but ORC2-like were only detected in posterior HMM searches using custom profiles created from the subunits identified in the initial searches (see SI Methods). We also extended the presence of ORC4-like to other Discoba and Metamonada (Table S1). Remarkably, we also detected potential ORC5 and ORC3 sequences in *Trichomonas vaginalis* (Metamonada), which indicates that this Excavata potentially has at least one copy of every ORC subunit. Moreover, *T. vaginalis* is the only Excavata with CDC6/ORC1 sequences branching within the ORC1 clade and not in the uncertain CDC6/ORC1 clade (Fig. S9).

The inclusion of a broadest taxon sampling was critical to reconstruct the hypothetical ancestral ORC subunit configuration of Excavata, as secondary losses were rampant in this group. According to the scenario proposed in Fig. 2, ORC2 would have been lost in *Leishmania braziliensis*, *Strigomonas culicis*, *Trypanosoma cruzi*, *Bodo saltans*, *Perkinsela sp.*, *Naegleria gruberi*, in the lineage leading to *Leptomonas seymouri* and *Leptomonas pyrrhocoris,* and in the lineage leading to *Spironucleus salmonicida* and *Giardia*. ORC3 would have been lost in *Monocercomonoides sp.* and in the lineage leading to Discoba and in the lineage leading to *S. salmonicida* and *Giardia*. ORC4 would have been lost in *Monocercomonoides sp.*, *Perkinsela sp.* and *S. salmonicida*. ORC5 would have been lost in *Perkinsela sp.* and in the lineage leading to *S. salmonicida* and *Giardia*. The scenario shown in Fig. 2 also proposes a loss of a CDC6/ORC1 gene in the root of Excavata. Some excavates have two copies, which we propose to be explained by lineage-specific duplications (in *Trichomonas vaginalis* and in the common ancestor of *Bodo saltans* and *Leishmania major*).

Our analyses show that ORC subunits are widely distributed in Amorphea and Diaphoretickes (Fig. S10A). Thus, any scenario in which Metamonada and Discoba were sister groups to either Amorphea or Diaphoretickes (H1 scenario in Fig. S10B) imply that CDC6+ORC1-5 were all present in the LECA (last eukaryotic common ancestor). Otherwise, there is more contingency surrounding the origin of CDC6+ORC1-5 (H2-H4 scenarios). Under the H2-H4 scenarios, if we assume the presence of ORC1-5 orthologs in Metamonada and also of ORC2, ORC4 and ORC5 in Discoba (Fig. S10A, ‘Possibly present’), it is plausible to propose that (i) CDC6/ORC1+5 were in the LECA, (ii) ORC1 and ORC3 were lost in the root of Discoba, and (iii) uncertain CDC6/ORC1 sequences from Excavata could correspond to highly diverged CDC6 sequences (H2A-H4A scenarios). Alternatively, because of a lack of phylogenetic resolution, the divergent sequences from Metamonada could have been misclassified as ORC1 and ORC3, and CDC6/ORC1 and ORC4-like sequences from Excavata could instead correspond to pre-diversified CDC6/ORC1 and ORC3/ORC4 genes (Fig. 1A). In such a case, the LECA could have had a pre-diversified ORC, consisting in CDC6/ORC1, ORC2, ORC3/4 and ORC5; which would have been fully diversified in the root of Amorphea and Diaphoretickes (H2B-H4B scenarios). However, even in these scenarios (H2-H4), in which Metamonada and Discoba appear as sister-group to all other eukaryotes, we find more parsimonious that ORC1, CDC6 and ORC3 were already present in the LECA and secondarily lost in some excavates (H2A-H4A scenarios). The reason is that ORC3 is also absent in many Amorphea and Diaphoretickes (Fig. S10A), and is the less abundant subunit in eukaryotes (2, 0.9, 0.75, 0.94 and 0.9 average copies per genome of CDC6/ORC1, ORC2, ORC3, ORC4 and ORC5, respectively). Losses of CDC6 or ORC1 are also observed in distinct eukaryotic lineages as, for example, Entamoeba or Apicomplexa (Fig. 2A). We thus conclude that the LECA probably had an already diversified ORC, although we cannot rule out alternative scenarios given the high divergence level of the potential orthologs found in Metamonada and Discoba.

**References**

Adl SM, Bass D, Lane CE, Lukes J, Schoch CL, Smirnov A, Agatha S, Berney C, Brown MW, Burki F, et al. 2019. Revisions to the Classification, Nomenclature, and Diversity of Eukaryotes. J Eukaryot Microbiol 66:4-119.

Cavalier-Smith T. 2010. Kingdoms Protozoa and Chromista and the eozoan root of the eukaryotic tree. Biol Lett 6:342-345.

Godoy PD, Nogueira-Junior LA, Paes LS, Cornejo A, Martins RM, Silber AM, Schenkman S, Elias MC. 2009. Trypanosome prereplication machinery contains a single functional orc1/cdc6 protein, which is typical of archaea. Eukaryot Cell 8:1592-1603.

Marques CA, Tiengwe C, Lemgruber L, Damasceno JD, Scott A, Paape D, Marcello L, McCulloch R. 2016. Diverged composition and regulation of the Trypanosoma brucei origin recognition complex that mediates DNA replication initiation. Nucleic Acids Res 44:4763-4784.

Tiengwe C, Marcello L, Farr H, Gadelha C, Burchmore R, Barry JD, Bell SD, McCulloch R. 2012. Identification of ORC1/CDC6-interacting factors in Trypanosoma brucei reveals critical features of origin recognition complex architecture. PLoS One 7:e32674.
