# Supplementary material for: Origin Recognition Complex (ORC) Evolution Is Influenced by Global Gene Duplication/Loss Patterns in Eukaryotic Genomes
Source: Genome Biol Evol. 2020 Jan 28;12(2):3878–89. doi: 10.1093/gbe/evaa011 (PMC7058166; doi:10.1093/gbe/evaa011)
Supplement: evaa011_Supplementary_Data [file evaa011_supplementary_data.zip › Ocaña-Pallarès Suppl Table 2.doc]

Supplementary Table 2. Primers used in this study

| **Name** | **Sequence** | **Notes** |
| --- | --- | --- |
| pNR-AvrII F | cctaggTCAACAAGCCCAAGCATCTAGATAT | Cloning of *NITRATE REDUCTASE* promoter |
| pNR-NdeI R | catatgGAGGGTCGTAAAGCTAATCAAGTGAA |  |
| CrORC1-F | CCTGCGCGGCTTCCACTT | qPCR |
| CrORC1-R | CCACCTGCACCTCGCCCTT |  |
| CreORC1_amiR_1F | ctagtTATTAAGGCAGGCTTTGGCGAtctcgctgatcggcaccatgggggtggtggtgatcagcgctaTCGCGAAAGCCTGCCTTAATAg | CreORC1amiR_1 |
| CreORC1_amiR_1R | ctagcTATTAAGGCAGGCTTTCGCGAtagcgctgatcaccaccacccccatggtgccgatcagcgagaTCGCCAAAGCCTGCCTTAATAa |  |
